# Minimal protein-only RNase P structure reveals insights into tRNA precursor recognition and catalysis

**DOI:** 10.1016/j.jbc.2021.101028

**Published:** 2021-07-31

**Authors:** Takamasa Teramoto, Takeshi Koyasu, Naruhiko Adachi, Masato Kawasaki, Toshio Moriya, Tomoyuki Numata, Toshiya Senda, Yoshimitsu Kakuta

**Affiliations:** 1 Laboratory of Biophysical Chemistry, Department of Bioscience and Biotechnology, Faculty of Agriculture, Kyushu University, Nishi-ku, Fukuoka, Japan; 2 Structural Biology Research Center, Institute of Materials Structure Science, High Energy Accelerator Research Organization (KEK), Tsukuba, Ibaraki, Japan; 3 Department of Materials Structure Science, School of High Energy Accelerator Science, The Graduate University of Advanced Studies (Soken-dai), Tsukuba, Ibaraki, Japan; 4 Laboratory of Biochemistry, Department of Bioscience and Biotechnology, Faculty of Agriculture, Kyushu University, Nishi-ku, Fukuoka, Japan

**Keywords:** molecular evolution, ribonuclease P (RNase P), precursor tRNA (pre-tRNA), cryo-EM, oligomerization, AaSelA, *A. aeolicus* L-seryl-tRNA^Sec^ selenium transferase, AtPRORP1, *Arabidopsis thaliana* PRORP1, HARPs, homologs of *Aquifex* RNase P/hexagram-like assembly proteinaceous RNase P, PPR, pentatricopeptide repeat, pre-tRNAs, precursor tRNAs, PrH, protruding helical, PRORP, protein-only RNase P, RNP, ribonucleoprotein

## Abstract

Ribonuclease P (RNase P) is an endoribonuclease that catalyzes the processing of the 5′ leader sequence of precursor tRNA (pre-tRNA). Ribonucleoprotein RNase P and protein-only RNase P (PRORP) in eukaryotes have been extensively studied, but the mechanism by which a prokaryotic nuclease recognizes and cleaves pre-tRNA is unclear. To gain insights into this mechanism, we studied homologs of *Aquifex* RNase P (HARPs), thought to be enzymes of approximately 23 kDa comprising only this nuclease domain. We determined the cryo-EM structure of Aq880, the first identified HARP enzyme. The structure unexpectedly revealed that Aq880 consists of both the nuclease and protruding helical (PrH) domains. Aq880 monomers assemble into a dimer via the PrH domain. Six dimers form a dodecamer with a left-handed one-turn superhelical structure. The structure also revealed that the active site of Aq880 is analogous to that of eukaryotic PRORPs. The pre-tRNA docking model demonstrated that 5′ processing of pre-tRNAs is achieved by two adjacent dimers within the dodecamer. One dimer is responsible for catalysis, and the PrH domains of the other dimer are responsible for pre-tRNA elbow recognition. Our study suggests that HARPs measure an invariant distance from the pre-tRNA elbow to cleave the 5′ leader sequence, which is analogous to the mechanism of eukaryotic PRORPs and the ribonucleoprotein RNase P. Collectively, these findings shed light on how different types of RNase P enzymes utilize the same pre-tRNA processing.

tRNA is a small RNA molecule that plays a central role in protein translation. The tRNA molecule must undergo its corresponding maturation process from precursor tRNAs (pre-tRNAs) to matured tRNA to exert its essential function (1, 2). In an early step in pre-tRNA maturation, the 5′ leader sequence is cleaved by the ribonuclease RNase P, which is a ubiquitous endoribonuclease in nature. However, two different forms exist. The first is the ribonucleoprotein (RNP) complex, composed of a single catalytic RNA component and a variable number of proteins. The RNA component possesses a catalytic center and plays a key role in pre-tRNA recognition (3, 4, 5, 6). The other form exists as protein-only RNase P (PRORP). The eukaryotic PRORP form is an enzyme of approximately 60 kDa comprising the pentatricopeptide repeat (PPR) domain, a central linker domain, and a nuclease domain (7, 8, 9). The N-terminal PPR domain is involved in pre-tRNA elbow recognition, whereas the C-terminal nuclease domain is responsible for the catalysis of 5′ leader cleavage. A comprehensive analysis of the PIN superfamily revealed that the nuclease domain of eukaryotic PRORPs belongs to the PIN superfamily (10). Recently, minimal PRORP (approximately 23 kDa) has been reported in bacteria and archaea (11). This type was initially found in the hyperthermophilic bacterium *Aquifex aeolicus* (gene name: *aq880*). A previous study reported that Aq880 comprises only the nuclease domain, which also belongs to the PIN superfamily (12). Bioinformatics analysis has shown that homologs of Aq880 are widely present in some phyla of bacteria and archaea (termed HARPs for homologs of *Aquifex* RNase P) (11, 13).

Because the different types of RNase P enzymes catalyze identical reactions, RNase P enzymes are an excellent target for understanding the diversity and molecular evolution of enzymes. Biochemical and structural analyses have provided tremendous knowledge regarding RNP RNase P. The structures of bacterial, archaeal, and eukaryotic RNP RNase P in complex with tRNA have elucidated the structural basis for the catalytic mechanism and pre-tRNA recognition of RNP RNase P (3, 4, 5, 6). The RNA component recognizes the elbow region and cleaves the 5′ leader sequence of the pre-tRNA molecule. Structures of eukaryotic PRORPs are also becoming evident (7, 9, 14, 15, 16, 17). The structure of the PPR domain in complex with tRNA elucidated the mechanism by which eukaryotic PRORP recognizes tRNA (9). Both RNP RNase P and eukaryotic PRORP recognize the structurally conserved elbow region of pre-tRNA, thereby measuring a precise and invariant distance from the tRNA elbow to cleave the 5′ leader sequence of pre-tRNA (9, 18).

In contrast, HARPs have only recently been discovered (11), and no structural information for them is currently available. According to the amino acid sequence of HARPs, they do not appear to contain a pre-tRNA-binding domain, such as the PPR domain of eukaryotic PRORPs. However, HARPs can catalyze the endonucleolytic maturation of pre-tRNA and substitute for RNP RNase P activity in *Escherichia coli*, as well as in *Saccharomyces cerevisiae* (11, 19). How HARPs specifically recognize pre-tRNA molecules and cleave the 5′ leader sequence of pre-tRNA is unknown.

To elucidate the structural basis for the catalytic mechanism and tRNA recognition of HARPs, we determined the cryo-EM structure of Aq880, the first HARP enzyme identified. The structure of Aq880 reveals a structural basis for pre-tRNA recognition and processing by Aq880, which is analogous to that of eukaryotic PRORPs.

## Results

### Pre-tRNA processing assay of Aq880

Recombinant Aq880 was tested for RNase P-specific cleavage (Fig. S1). *Arabidopsis thaliana* PRORP1 (AtPRORP1), used as a control for activity and 5′ tRNA product, was previously shown to cleave the pre-tRNA at the canonical site (20). Aq880 can cleave the pre-tRNA that contains a 9-nt 5′ leader sequence at the same site as AtPRORP1 (Fig. S1). To further analyze metal dependence, we examined whether Aq880 can catalyze pre-tRNA cleavage with Mg^2+^ and EDTA. The results showed that Aq880 with Mg^2+^ could catalyze pre-tRNA cleavage, whereas no cleavage activity was observed upon the addition of EDTA (Fig. S1). Mg^2+^ is likely to be essential for Aq880 processing activity, similar to other types of RNase P.

### Cryo-EM single-particle analysis of Aq880

**Figure 1:**
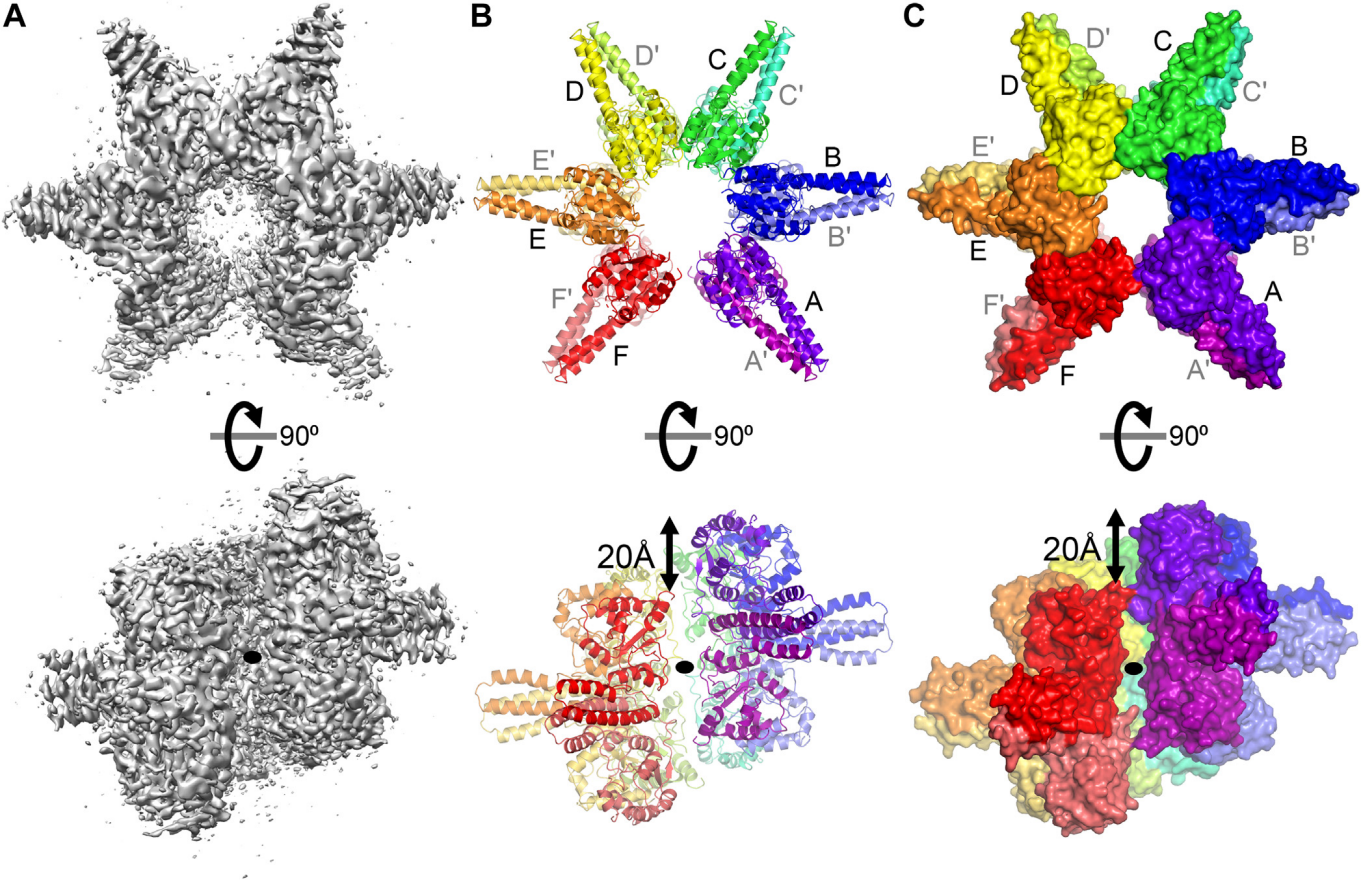
**Dodecameric assembly of Aq880.** The cryo-EM map reconstruction (*A*) of Aq880 and its corresponding models are shown as a cartoon (*B*) and a surface (*C*). The 12 subunits are shown in different colors. *Black ovals* indicate the axis of C2 symmetry. The 12 subunits are labeled A, A', B, B', C, C', D, D', E, E', F, and F', respectively.

We determined the structure of Aq880 using a single-particle cryo-EM. Over 2000 micrographs were collected from grids prepared with recombinant Aq880. The micrograph showed hexagram-like particles of approximately 10 nm (Fig. S2). After 3D classification, the particles converged into a single prominent class with a resolution of 3.62 Å (Fig. 1*A* and Fig. S3). The cryo-EM reconstruction of Aq880 revealed a dodecameric assembly (Fig. 1). The final built protein ranged from Met1 to His190, except for four low-density residues, namely Asp23, Gln24, Arg191, and Phe192 (Fig. S4).

**Figure 2:**
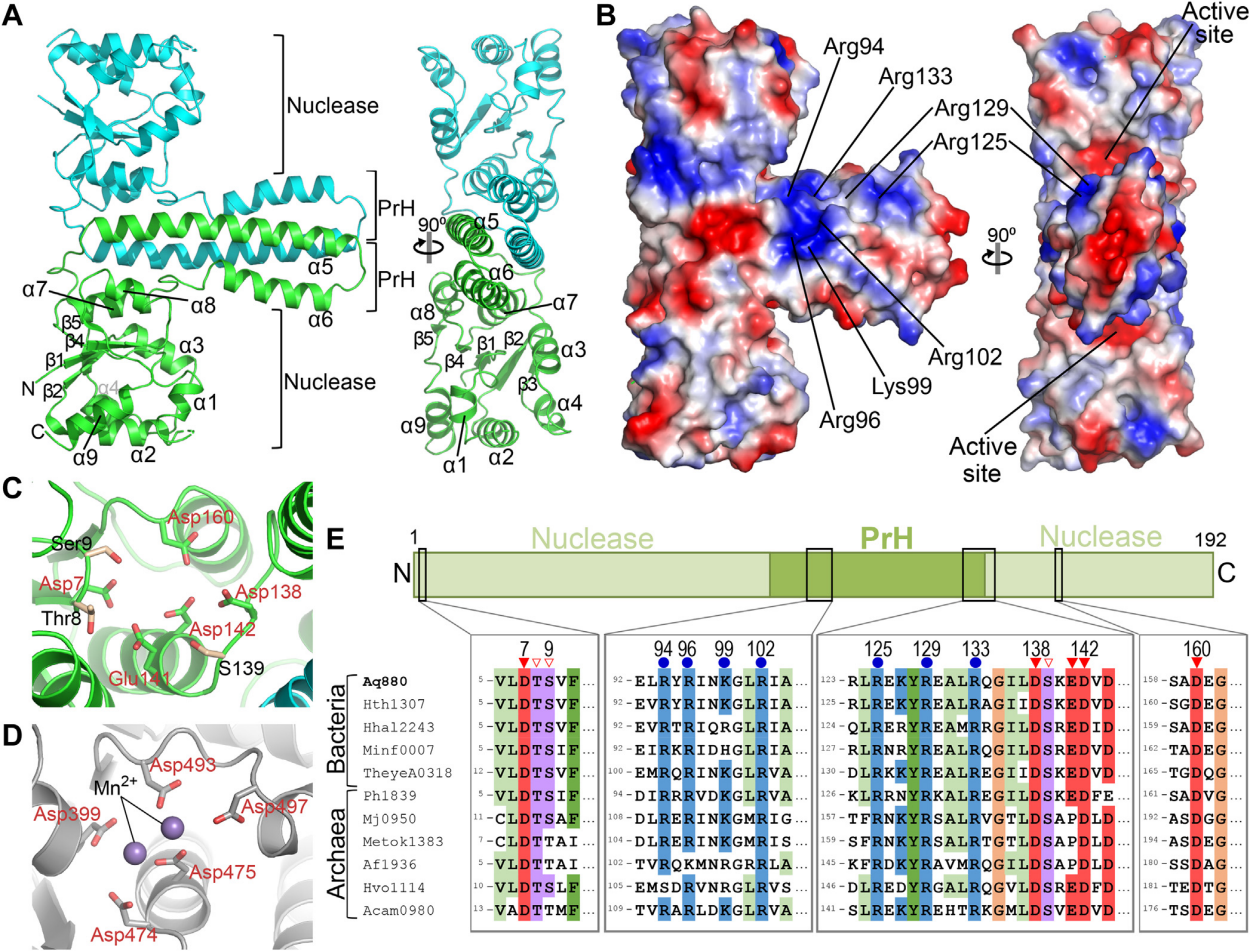
**Dimer structure of Aq880.***A*, the dimer structure of Aq880 is shown as a cartoon representation. *B*, the electrostatic surface potential of Aq880 dimer. *Blue* surfaces indicate positive charges, whereas *red* surfaces indicate negative charges. The basic residues in the PrH domain, which are conserved among HARPs, are shown. *C*, the active site of Aq880. Residues in the active site of Aq880 are shown as *stick* models. *D*, the active site of AtPRORP1 (PDB ID: 4G24). Residues in the active site of AtPRORP1 are shown as *stick* and *sphere* models, respectively. *E*, amino acid sequence alignment of the active site and the conserved positively charged site from HARPs. The domain architecture of Aq880 is shown above with their corresponding amino acid residue boundaries. *Red closed triangles* and *open triangles* indicate the acidic residues and the other residues, respectively, in the active site of Aq880. *Blue closed circles* indicate the positively charged residues in the PrH domain of Aq880. AtPRORP1, *Arabidopsis thaliana* PRORP1; HARPs, homologs of *Aquifex* RNase P/hexagram-like assembly proteinaceous RNase P; PrH, protruding helical.

The cryo-EM structure revealed that Aq880 is a homododecamer (Fig. 1, *B* and *C*). Each subunit has a typical PIN-like fold, which comprises a single α/β/α domain with a five-stranded parallel β-sheet (β1-β5) surrounded by nine α-helices (α1-α9) (Figs. 2*A* and S4). Notably, each subunit also possesses two prominent helices (α5 and α6) that are inserted into the nuclease domain (Figs. 2*A* and S4). These unique helices correspond to the protruding part of the hexagram in the micrograph. Hence, the region of the two helices was denoted as the “protruding helical” (PrH) domain. The structure also revealed that the subunit of Aq880 consists of two domains, the nuclease and PrH domains. A search for structural homologs of the nuclease domain of Aq880 using the Dali server (21) revealed structural similarities with the VapC4 toxin from *Pyrococcus horikoshii* (PDB ID: 5H4G) (Fig. S5). The VapC4 toxin is a well-known ribonuclease belonging to the VapC group of the PIN superfamily (22), indicating that the nuclease domain of Aq880 is responsible for cleavage catalysis.

### Dodecameric assembly of Aq880

The cryo-EM structure revealed that Aq880 forms a homododecamer. Aq880 was eluted at 264 kDa by analytical gel filtration chromatography, which corresponds to the molecular weight (270 kDa) of the Aq880 dodecamer (monomer: 22.5 kDa) (Fig. S6*a*). To further confirm the oligomeric state in solution, we measured the particle size of Aq880. The analysis showed that the particle size of Aq880 was 7 to 11 nm (Fig. S6*b*), which is consistent with the cryo-EM micrograph of Aq880. These results indicate that Aq880 exists as a dodecameric assembly in solution.

The dodecameric assembly of Aq880 showed a left-handed one-turn superhelical structure with six dimers (Fig. 1, *B* and *C*). The first (A/A′) and last (F/F′) dimers were shifted by 20 Å in one turn (Fig. 1, *B* and *C*). The two subunits formed a dimer mediated by the PrH domain, consisting of α5 and α6 (Fig. 2*A*) (Movie S1). The dimer interface is mainly comprised of a hydrophobic environment (Fig. S7*a*). Sequence comparison of HARPs revealed that the residues of the dimer interface are highly conserved among HARPs (Fig. S7*b*). Six dimers formed an oligomeric assembly mediated mainly by the nuclease domain. Each dimer was aligned side-by-side with β-sheet interactions to form a hexamer of the dimer (Fig. S8) (Movie S1). The interface between the two dimers contained side-chain interactions, including electrostatic (Arg75/Asp153) and aromatic stacking (Phe77/Pro82) interactions (Fig. S8). However, these residues including Arg75, Phe77, and Asp153 were less conserved among HARPs. This indicates that the β-sheet main-chain interaction is a dominant contributor to the formation of the hexamer of dimers.

To determine whether the dodecameric assembly is a common feature of HARPs, we analyzed the cryo-EM image of *Hydrogenobacter thermophilus* HARP enzyme, Hth1307, which shares an amino acid sequence identity of 67% with Aq880 (Fig. S4). The 2D classification of the particle images of Hth1307 showed hexagram-like superhelical structure particles identical to Aq880 (Fig. S9). Thus, we confirmed that HARPs commonly form a dodecameric assembly. Therefore, we propose HARP to stand for hexagram-like assembly proteinaceous RNase P, instead of the homolog of *Aquifex* RNase P.

### Active site of Aq880

In a previous study, four aspartate residues (Asp7, Asp138, Asp142, and Asp160) were found to be essential for the nuclease activity of Aq880 (11, 12). In the present study, the structure of Aq880 revealed that these aspartate residues are localized in the nuclease domain near the PrH domain (Fig. 2, *A* and *C*), representing the active site of Aq880. In addition, the active site contained the Thr8, Ser9, Ser139, and Glu141 residues (Fig. 2*C*). All eight residues in the active site were found to be highly conserved among HARPs (Fig. 2*E*), suggesting that they play a crucial role in nuclease activity and specificity.

Both HARPs and eukaryotic PRORPs have a PIN-like nuclease domain that catalyzes identical reactions. The PIN superfamily of nucleases is characterized by the presence of four or five key acidic residues at the active site. The structural analysis of AtPRORP1 revealed that its active site contains five acidic residues (Asp399, Asp474, Asp475, Asp493, and Asp497) and two coordinated metal ions (Mn^2+^) (Fig. 2*D*). A comparison of the active sites between Aq880 and AtPRORP1 revealed that the five acidic residues were structurally conserved (Fig. 2, *C* and *D*). As for metal ions, despite the addition of excess magnesium to the protein solution, we did not observe a discrete density for a metal ion in the active site of Aq880. The structure of Aq880 revealed that the active site of Aq880 contains five acidic residues required for catalysis, which is analogous to the active site of eukaryotic PRORPs. Therefore, we propose that HARPs and eukaryotic PRORPs have similar catalytic mechanisms.

### Model of the Aq880–pre-tRNA complex

We determined the cryo-EM structure of Aq880; however, our attempts to determine the structure in complex with pre-tRNA were unsuccessful, leaving the question of how Aq880 recognizes substrate pre-tRNA molecules unanswered. Structural analyses revealed that RNP RNase P recognizes both the elbow region and the 5′ cleavage site of the pre-tRNA molecule, suggesting that the enzyme is preconfigured to measure a fixed distance (approximately 42 Å) on pre-tRNA as a molecular ruler. Eukaryotic PRORPs are also likely to measure the distance via elbow recognition by their PPR domain. Elbow recognition by the AtPRORP1 PPR domain utilizes clustered basic residues (9).

**Figure 3:**
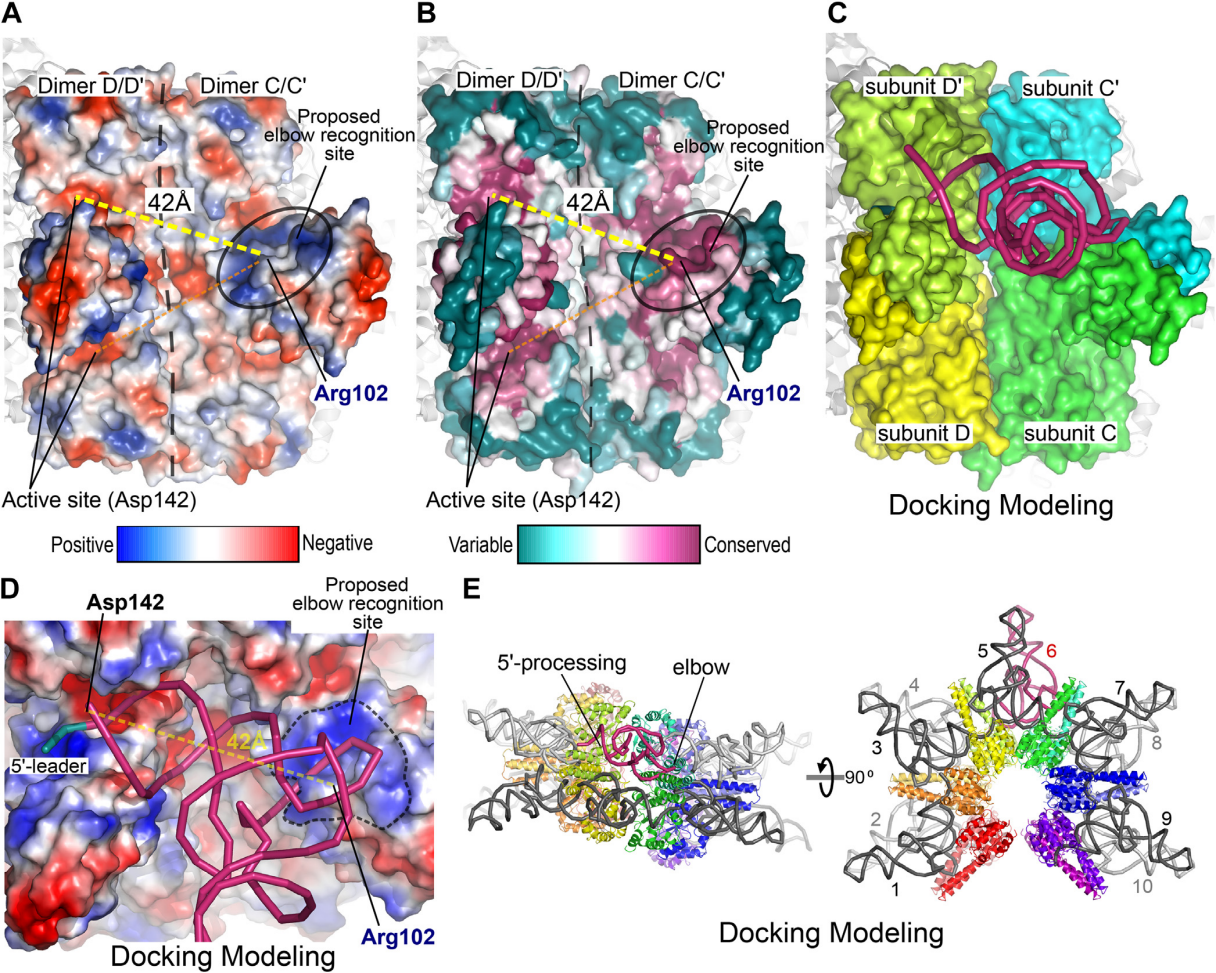
**Model of pre-tRNA recognition and catalysis by HARPs.***A* and *B*, electrostatic surface potential (*A*) and surface colored by degree of conservation (*B*) of two adjacent Aq880 dimers. *Dashed lines* indicate the distance between the active site and the conserved positively charged site of Aq880. The ConSurf server (36) was used to identify sequence homologs and project the degree of conservation on the structure of Aq880. *C*, docking pre-tRNA onto two adjacent Aq880 dimers. *D*, a close-up view of the potential interface between the pre-tRNA and the Aq880. *E*, a docking model of the Aq880 dodecamer with ten docked tRNA molecules. The tRNA molecules are shown as *ribbon* models. The representative tRNA molecule is shown in *pink*. HARPs, homologs of *Aquifex* RNase P/hexagram-like assembly proteinaceous RNase P; pre-tRNAs, precursor tRNAs.

Considering these findings, we searched for the elbow recognition site of the Aq880 dodecamer using two criteria: (i) 42 Å away from the active site and (ii) a cluster of conserved basic residues. We found a positively charged site composed of many basic residues (Arg94, Arg96, Lys99, Arg102, Arg125, Arg129, and Arg133) (Figs. 2*B* and 3*A*). This site was formed across the PrH domains of the two subunits of one dimer. Arg94, Arg96, Lys99, and Arg102 were derived from the helix α5 of one subunit, whereas Arg125, Arg129, and Arg133 were derived from the helix α6 of the other subunit. These basic residues were highly conserved among HARPs (Figs. 2*E* and 3*B*). The distances from the center of positively charged site (Arg102) to the two centers of active sites (Asp142) of the dimer are 22 Å and 25 Å, respectively, whereas the distances from the site to the two active sites of the neighboring dimer were 42 Å and 40 Å, respectively (Fig. 3, *A* and *B*). As these satisfy the two criteria, these positively charged regions may be used for elbow recognition. To confirm these possibilities, we docked a pre-tRNA molecule (PDB ID: 6AH3) onto the dimer C/C′ and dimer D/D′ of Aq880 structure in two different orientations (Fig. 3). The docking models showed that one candidate space between the elbow recognition sites and the active site could fit the pre-tRNA molecule for recognition and catalysis (Fig. 3, *C* and *D*). The other candidate space could not accommodate the tRNA molecule because of steric hindrances (Fig. S10). In the docking model, the pre-tRNA molecule interacts with the three subunits (subunit C, C′, and D′) of Aq880 (Fig. 3*C* and Movie S2). Dodecamers possess 12 conserved positively charged sites. Because the first (A/A′) and the last (F/F′) dimers are shifted by 20 Å (Fig. 1), the two terminal sites cannot be used for elbow recognition. Hence, using the same docking approach, a dodecamer of Aq880 can accommodate ten molecules of pre-tRNA (Fig. 3*E* and Movie S3). We propose that the superhelical structure can contribute to formation of pre-tRNA-binding space. Our docking models provide an initial structural insight into how the 23-kDa nuclease acquires substrate specificity by oligomerization.

## Discussion

Here, we present the cryo-EM structure of dodecameric assembly of Aq880. The dodecameric assembly of Aq880 is reminiscent of the decameric assembly of *A. aeolicus* L-seryl-tRNA^Sec^ selenium transferase (AaSelA) (gene name: *aq1031*). The crystal structure revealed that AaSelA forms a ring-shaped homodecamer that binds ten tRNA^Sec^ molecules, and four AaSelA subunits are involved in tRNA recognition and catalysis (Fig. S11) (23). This AaSelA tRNA binding by oligomerization is consistent with the idea that Aq880 recognizes pre-tRNA by oligomerization. The crystal structure of AaSelA in complex with tRNA^Sec^ supports our pre-tRNA-binding model of Aq880. *A. aeolicus* is known to possess a small, compact genome with a reduced number of genes (24). Owing to limitations in the genome size, *A. aeolicus* seems to utilize small proteins to generate oligomers, thereby obtaining a diverse function.

The docking model suggests that Aq880 recognizes the elbow region of pre-tRNA by several basic residues. Other elbow-binding enzymes, AaSelA and *Bacillus subtilis* RNase Z, also recognize phosphate backbones of the elbow region by basic residues (Fig. S12, *a–c*) (23, 25). In these elbow-binding enzymes, electrostatic interaction is the dominant contributor to elbow recognition. AtPRORP1 and *Archaeoglobus fulgidus* CCA-adding enzyme utilize aromatic residues for stacking interaction with bases of tRNA in addition to electrostatic interaction by basic residues (Fig. S12, *d* and *e*) (9, 26). *Thermotoga maritima* RNP RNase P and *B. subtilis* T-box riboswitches recognize the elbow region predominantly through base–base stacking interaction (Fig. S12, *f* and *g*) (4, 27). These structures indicate that the elbow recognition has a strong component of aromatic stacking. Therefore, we investigated whether aromatic residues could be involved in elbow recognition. Two tyrosine residues, Tyr95 and Tyr128, are present near the elbow recognition site. Tyr95 is exposed on the protein surface but is not conserved among HARPs (Fig. 2*E*). Tyr128 is highly conserved (Fig. 2*E*), but is not exposed on the protein surface, suggesting that Tyr128 plays an important role in dimer formation. (Fig. S7*c*). These suggest that aromatic residues are unlikely to be involved in elbow recognition by Aq880. Although there is a slight difference in the recognition mode, the elbow region is a hotspot for substrate recognition by enzymes (Fig. S12).

**Figure 4:**
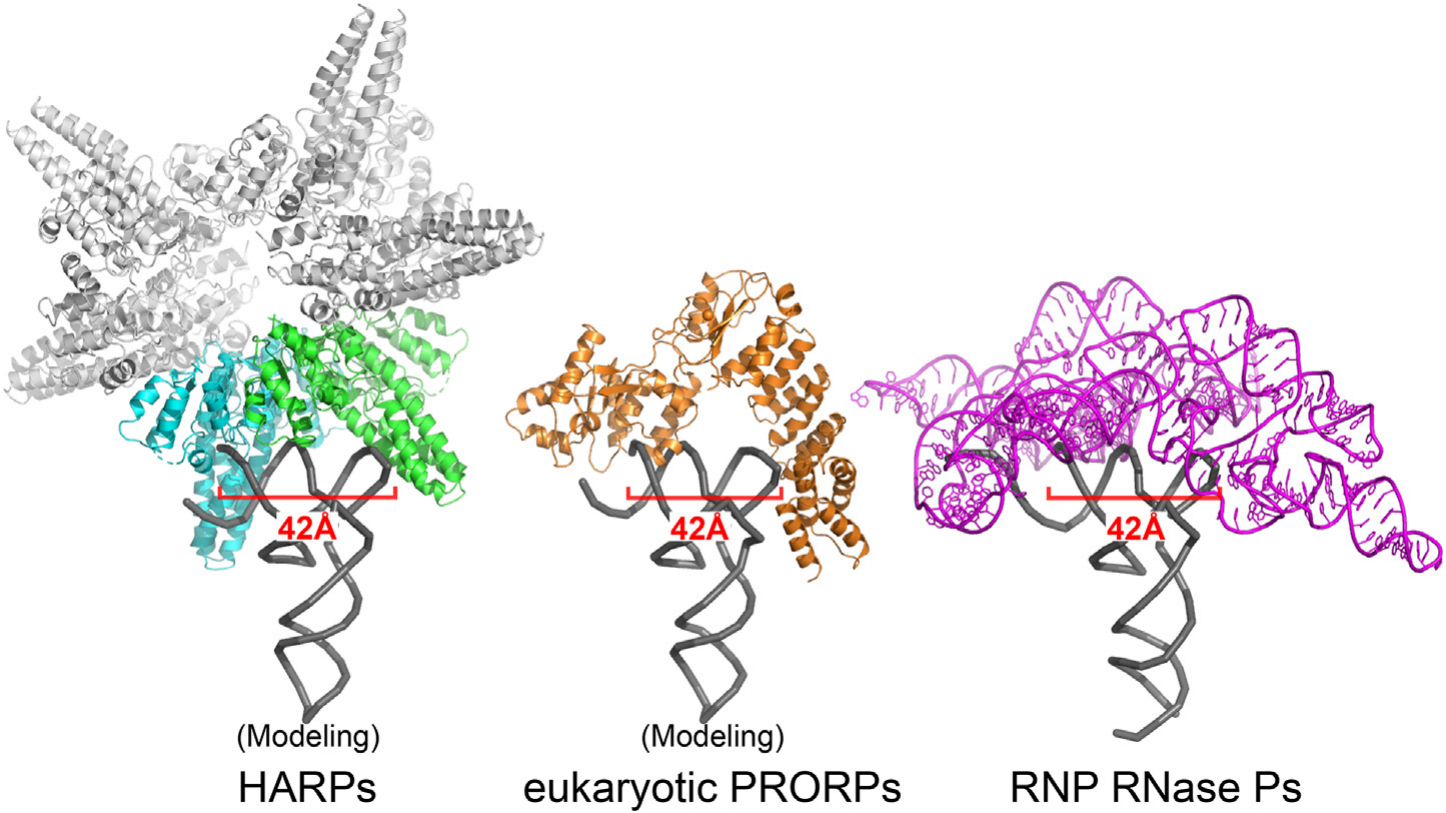
**All types of RNase P function as molecular rulers.** HARP and eukaryotic PRORP structures in complex with tRNA are docking models. The structure of RNP RNase P in complex with tRNA is modified from the determined structure (PDB ID: 3Q1Q). HARPs, homologs of *Aquifex* RNase P/hexagram-like assembly proteinaceous RNase P; PRORP, protein-only RNase P.

Eukaryotic PRORPs could function as molecular rulers by fusing the PPR domain to the nuclease domain in the appropriate positions. In contrast, HARPs seem to serve as molecular rulers by appropriately positioning the PrH domain relative to the nuclease domain by oligomerization. Our study can convincingly explain how the small ribonuclease HARPs oligomerize to specifically recognize the invariant distance between the elbow and the 5′ cleavage site, allowing for the site-specific cleavage of pre-tRNA. All types of RNase P can measure the invariant distance between the elbow region and the 5′ cleavage site, indicating convergence of the same solution for pre-tRNA processing (9, 18) (Fig. 4).

Our structure is consistent with the structure of HARP from *Halorhodospira halophila* (Hhal2243) by cryo-EM, reported in a concurrent bioRxiv preprint (28). Hhal2243 shares an identity of 53% of amino acid sequence with Aq880. In agreement with our findings, the study reports that Hhal2243 forms a homododecamer with a left-handed one-turn superhelical structure, and dodecameric assembly is necessary for catalysis. Their mutational analysis also demonstrated that a cluster of basic residues in the PrH domain is required for pre-tRNA processing. This strongly supports our recognition mechanism for HARPs. However, further structural analysis of the pre-tRNA complex is required to understand the details of the pre-RNA processing mechanism.

## Experimental procedures

### Protein expression and purification

The cDNA sequences encoding Aq880 (residues 1–192) and Hth1307 (residues 1–194) were obtained from a gene synthesis service (Thermo Fisher Scientific) and then subcloned into the pE_SUMO vector, which encodes an N-terminal His_6_-SUMO tag. Recombinant Aq880 and Hth1307 were expressed in the *E. coli* strain BL21-CodonPlus (DE3)-RIL (Agilent Technologies). The proteins were first purified by Ni-NTA agarose chromatography (Qiagen), and then the eluted proteins were digested by Ulp1 to remove the SUMO tag, followed by purification on a HiTrap Q column (Cytiva). The peak fractions containing target proteins were pooled and concentrated. The proteins were further purified using a HiLoad 16/60 Superdex 200 column (Cytiva). The peak fractions were pooled and concentrated to 5 mg/ml. Further details are provided in supporting information.

### Cryo-EM sample preparation and data collection of Aq880

For cryo-grid preparation, 3 μl of the sample of Aq880 (5 mg/ml) in 50 mM Tris HCl (pH 8.0), 100 mM NaCl, and 0.5 mM TCEP was applied onto a holey carbon grid. The grid was blotted and then flash-frozen in liquid ethane using Vitrobot Mark IV (Thermo Fisher Scientific). Micrographs were acquired on a Talos Arctica (Thermo Fisher Scientific) microscope operating at 200 kV. The movie micrographs were collected on a 4k × 4k using a Falcon 3EC direct electron detector at a nominal magnification of 120,000 (0.88 Å/pixel). Forty-eight movie fractions were recorded at an exposure of 1.04 electrons per Å^2^. The defocus steps used were ‒1.0, ‒1.5, ‒2.0, and ‒2.5 μm. Further details are provided in supporting information.

### Cryo-EM data processing of Aq880

The dataset was processed using RELION-3.1 (29). Dose-fractionated movies were gain-normalized, aligned, and dose-weighted using RELION's implementation (29). The contrast transfer function was determined using the Gctf program (30). A total of 2903 particles were manually picked and used as a 2D reference that was subsequently used to automatically pick the set of 2370 micrographs. A total of 1,486,899 candidate particles were extracted and cleaned using several cycles of reference-free 2D classification. Finally, the 238,017 particles belonging to the best-aligned particles were subsequently subjected to homogenous 3D refinement. The last 3D refinement (C2 symmetry, 240 Å mask diameter) with a soft-edged 3D mask, and post-processing generated the result at 3.62 Å resolution. Further details are provided in supporting information and summarized in Figures S2 and S3.

### Cryo-EM sample preparation, data collection, and data processing of Hth1307

Cryo-grid preparation, data collection, and data processing of Hth1307 for 2D classification were similar to those described above. Ten micrographs were obtained using a Talos Arctica microscope. A total of 550 particles were selected manually, extracted, and classified using a reference-free 2D classification (Fig. S9). Further details are provided in supporting information.

### Cryo-EM model building

A model was built manually for cryo-EM reconstruction. The building model was improved using a real-space refinement in Phenix (31) combined with iterative rounds of building in COOT (32). MolProbity (33) and Mtriage (34) were used to evaluate the model. The model statistics are listed in Table S1. Figures and videos were prepared using PyMOL (Schrödinger, LLC) and UCSF Chimera (35).

### Pre-tRNA processing assays

Processing assays were carried out in the reaction buffer (20 mM Tris HCl, pH 8.0, 100 mM NaCl, 1 mM TCEP), supplemented with 10 mM MgCl_2_ or 10 mM EDTA. Cleavage assays were performed with 1 μM Aq880 and 2.5 μM pre-tRNA at 37 °C for 30 min. The reaction solutions were subjected to electrophoresis on 15% TBE-urea gels. The gels were stained with SYBR Gold (Thermo Fisher Scientific). The pre-tRNA substrate and the 5′-matured product were visualized at 412 nm using a transilluminator.

### Analytical gel filtration analysis and particle-size analysis

The details are provided in supporting information.

## Data availability

The cryo-EM maps of Aq880 were deposited in the Electron Microscopy Data Bank under accession code EMD-31432. Structural coordinates were deposited at the Protein Data Bank under accession code 7F3E.

## Supporting information

This article contains supporting information.

## Conflict of interest

The authors declare that they have no conflicts of interest with the contents of this article.
