# Supplementary material for: Minimal protein-only RNase P structure reveals insights into tRNA precursor recognition and catalysis
Source: J Biol Chem. 2021 Jul 31;297(3):101028. doi: 10.1016/j.jbc.2021.101028 (PMC8405995; doi:10.1016/j.jbc.2021.101028)
Supplement: Supporting information [file mmc1.pdf]

Supporting Information

Minimal protein-only RNase P structure reveals insights into tRNA precursor recognition and catalysis

Takamasa Teramoto, Takeshi Koyasu, Naruhiko Adachi, Masato Kawasaki, Toshio Moriya, Tomoyuki Numata, Toshiya Senda, Yoshimitsu Kakuta

Protein expression and purification

The cDNA sequences encoding Aq880 (residues 1-192) and Hth1307 (residues 1-194) were obtained from a gene synthesis service (Thermo Fisher Scientific), and then subcloned into the pE_SUMO vector, which encodes an N-terminal His6-SUMO tag. The recombinant proteins were expressed in E. coli strain BL21-CodonPlus (DE3)-RIL (Agilent Technologies) at 18 °C overnight after induction with 0.5 mM IPTG. The cells were collected by centrifugation, and pellets were resuspended in lysis buffer (containing 50 mM Tris-HCl, pH 8.0, 500 mM NaCl) and stored at −80 °C until use. The cells were disrupted by sonication followed by centrifugation to remove cell debris. The soluble fraction was applied to a Ni-NTA agarose column and thoroughly washed with lysis buffer containing 20 mM imidazole. The target SUMO fusion protein was eluted with lysis buffer containing 400 mM imidazole. The fusion protein was cleaved overnight with 0.2 mg of Ulp1 protease and dialyzed against a buffer containing 50 mM Tris-HCl, pH 8.0, 100 mM NaCl and 0.5 mM TCEP. The protein was then loaded onto a HiTrap Q column (Cytiva). Bound proteins were eluted using a linear gradient from 0.1 to 1 M NaCl in 50 mM Tris-HCl, pH 8.0. Peak fractions containing Aq880 were pooled and concentrated and reducing agent was added (final concentration of 1 mM dithiothreitol). The protein was purified further using a HiLoad 16/60 Superdex 200 column (Cytiva), equilibrated with 50 mM Tris-HCl, pH 8.0, 100 mM NaCl, and 0.5 mM TCEP. Final purified protein was concentrated to 5 mg/mL.

Cryo-EM sample preparation, data collection

For cryo-grid preparation, 3 μL of the sample of Aq880 (5 mg/mL) in 50 mM Tris-HCl, pH 8.0, 100 mM NaCl, and 0.5 mM TCEP was applied onto a holey carbon grid (Quantifoil, Cu, R1.2/1.3, 300 mesh). The grid was rendered hydrophilic by a 30 sec glow-discharge in air (11 mA current) with PIB-10 (Vacuum Device Inc., Ibaraki, Japan). The grid was blotted for 5 sec (blot force 20) at 18°C and 100% humidity and then flash-frozen in liquid ethane using Vitrobot Mark IV (Thermo Fisher Scientific, Waltham, MA, USA). For automated data collection, 2,370 micrographs were acquired on a Talos Arctica (Thermo Fisher Scientific) microscope operating at 200 kV in nanoprobe mode using EPU software. The movie micrographs were collected on a 4k × 4k using a Falcon 3EC direct electron detector (electron counting mode) at a nominal magnification of 120,000 (0.88 Å/pixel). Forty-eight movie fractions were recorded at an exposure of 1.04 electrons per $Å^2$ per fraction corresponding to a total exposure of 50.00 e^-/Å2. The defocus steps used were –1.0, –1.5, –2.0, and –2.5 μm.

Cryo-EM data processing.

First, the movie fractions were aligned, dose-weighted, and averaged using RELION’s own implementation on 5 × 5 tiled fractions with a B-factor of 300. The non-weighted movie sums were

used for Contrast Transfer Function (CTF) estimation with the Gctf program. The dose-weighted sums were used for all subsequent steps of image processing. The subsequent processes of particle picking, two-dimensional (2D) classification, ab initio reconstruction, three-dimensional (3D) classification, 3D refinement, CTF refinement, and Bayesian polishing were performed using RELION-3.1. Initially, 2,903 particles were manually picked and performed 2D classification for preparing a 2D reference of template-matching-based auto-pick by RELION-3.1. From the 2,370 micrographs, 1,486,899 particles were automatically picked and extracted while rescaling to 2.64 Å/pixel with 100-pixel box size. The extracted particle images were split into 20 sets and subjected to the reference-free 2D classification for three cycles (200 expected classes, 160 Å mask diameter).

After the three cycles of reference-free 2D classification, the 510,382 particles corresponding to the best 39 classes, which had around 160 Å diameter and displayed secondary-structural elements, were selected from the result, and then used for ab initio reconstruction (asymmetry, single expected class, 180 Å mask diameter). C1 symmetry was imposed on the generated ab initio map, which was used as an initial 3D reference for the first 3D classification (4 expected classes, 220 Å mask diameter). The 3D volume and 304,342 particles of the best 3D class, which displayed the highest resolution, were used for the subsequent 3D refinements (C1 symmetry, 220 Å mask diameter). The refined volume and particle images were rescaled to 0.88 Å/pixel with a 300-pixel box size and used for the 3D refinement (C1 symmetry, 220 Å mask diameter). The generated 3D refined map and 304,342 particles was used for the first no-alignment 3D classification (4 expected classes, 220 Å mask diameter). The 3D volume, which displayed the highest resolution, was rescaled to 2.64 Å/pixel with 100-pixel box size and used as a 3D reference for the second 3D classification.

The 1,158,797 particles were selected from the results of reference-free 2D classification after the first cycle and used for the second 3D classification (4 expected classes, 200 Å mask diameter). The 3D volume and 538,117 particles of the best 3D class, which displayed the highest resolution, were used for the subsequent 3D refinements (C1 symmetry, 200 Å mask diameter). The refined volume and particle images were rescaled to 0.88 Å/pixel with a 300-pixel box size and used for the 3D refinement (C1 symmetry, 200 Å mask diameter). The generated 3D refined map was used for the second no-alignment 3D classification (2 expected classes, 200 Å mask diameter). The 3D volume and 238,017 particles of the best 3D class, which displayed the highest resolution, were used for the subsequent 3D refinement (C1 symmetry, 240 Å mask diameter). The cycle of CTF refinement and Bayesian polishing was repeated four times. The 3D refinement (C1 symmetry, 240 Å mask diameter) with a soft-edged 3D mask (15-pixel extension, 30-pixel soft cosine edge) was executed after each CTF refinement and Bayesian polishing step. Then, C_2 symmetry was imposed on the generated 3D refined map, which was used for the 3D

refinement (C2 symmetry, 240 Å mask diameter). The single CTF refinement was performed. The 3D refinement (C2 symmetry, 240 Å mask diameter) with a soft-edged 3D mask (15-pixel extension, 30-pixel soft cosine edge) was executed after CTF refinement step. The last 3D refinement (C2 symmetry, 240 Å mask diameter) with a soft-edged 3D mask (15-pixel extension, 30-pixel soft cosine edge) and post-processing generated the final result at 3.62 Å resolution.

For calculation of the global resolution estimation after each 3D refinement, the gold-standard Fourier Shell Correlation (FSC) resolution with a criterion of 0.143 was used. The local resolution was estimated using the implementation of RELION-3. The model-to-map FSC resolution with 0.5 criterion was calculated using phenix.mtriage. For the visualization of the output 3D images, UCSF Chimera was used.

Cryo-EM sample preparation, data collection, and data processing of Hth1307

For cryo-grid preparation, 3 μL of the sample of Hth1307 (5 mg/mL) in 50 mM Tris-HCl, pH 8.0, 100 mM NaCl, and 0.5 mM TCEP was applied onto a holey carbon grid (Quantifoil, Cu, R1.2/1.3, 300 mesh). The grid was rendered hydrophilic by a 30 sec glow-discharge in air (11 mA current) with PIB-10 (Vacuum Device Inc., Ibaraki, Japan). The grid was blotted for 5 sec (blot force 20) at 18°C and 100% humidity and then flash-frozen in liquid ethane using Vitrobot Mark IV (Thermo Fisher Scientific, Waltham, MA, USA). For automated data collection, 10 micrographs were acquired on a Talos Arctica (Thermo Fisher Scientific) microscope operating at 200 kV in nanoprobe mode using EPU software. The movie micrographs were collected on a 4k × 4k using a Falcon 3EC direct electron detector (linear mode) at a nominal magnification of 92,000 (1.13 Å/pixel). Twelve movie fractions were recorded with Volta Phase Plate at an exposure of 4.17 electrons per $Å^2$ per fraction corresponding to a total exposure of 50.00 e^{-}/Å2. The defocus used were –1.0 μm. The Hth1307 dataset was processed by RELION-3.1. Dose-fractionated movies were gain-normalized, aligned, and dose-weighted using RELION's own implementation. The contrast transfer function (CTF) was determined using the Gctf program. A total of 550 particles was manually picked. The 550 particles were extracted and classified by reference-free 2D classification (220 Å mask diameter).

Analytical gel filtration analysis

Gel filtration analysis was performed with a Superdex 200 increase 10/300 GL column (Citiva) equilibrated with 50 mM Tris-HCl, pH 8.0, 100 mM NaCl, and 0.5 mM TCEP. The standard proteins (thyroglobulin, ferritin, aldolase, conalbumin, ovalbumin, and carbonic anhydrase) (1 mg/mL) from gel filtration calibration kits (Cytiva) were used to prepare a calibration curve. The Aq880 protein (5 mg/mL) was loaded onto the column, and the molecular weight of Aq880 was determined from the calibration curve.

Particle size analysis

The average particle size and ζ-potential measurements of Aq880 were performed using a Zeta-potential & Particle size Analyzer ELSZ-2000 (Otsuka Electronics). The particle size and ζ-potential in multiple runs were analyzed using 200 μL of the sample (0.5 mg/mL).

Supplementary Table 1 cryo-EM data collection, refinement, and validation statistics.

(EMDB/PDB ID)	Aq880<br>(EMD-31432/7F3E)
**Data collection and processing**	
Microscope	Talos Arctica
Voltage (kV)	200
Detector	Falcon 3EC
Magnification	120,000
Pixel size (Å)	0.88
Automation software	EPU
Total exposure (e–/Å^2)	50
Exposure rate (e–/Å^2 fraction)	1.04
Number of frames	48
Defocus range (μm)	–1, –1.5, –2, –2.5
Number of collected micrograph	2,370
Number of particles for Class2D	1,486,899
Number of particles for Class3D	538,117
Number of particles for Refine3D	238,017
Symmetry imposed	C_2
Map resolution (Å)	3.62
FSC threshold	0.143
Map resolution range (Å)	3.40–5.05
**Refinement**	
Refinement programs	PHENIX/Coot
Map-to-model resolution (Å)	4.03
FSC threshold	0.5
Model resolution range (Å)	3.40–5.05
Model composition	
Non-hydrogen atoms	18564
Protein residues	2256
B factors (Å^2)	
Protein	15.7
Map-model CC	
CC (mask)	0.67
CC (box)	0.63
CC (peaks)	0.54
CC (volume)	0.64
R.m.s. deviations	
Bond lengths (Å)	0.005
Bond angles (°)	0.692
Validation	
MolProbity score	1.85
Clash score	12.2
Poor rotamer (%)	0.0
Ramachandran plot	
Favored (%)	96.2
Allowed (%)	3.8

Supplementary Fig. 1.

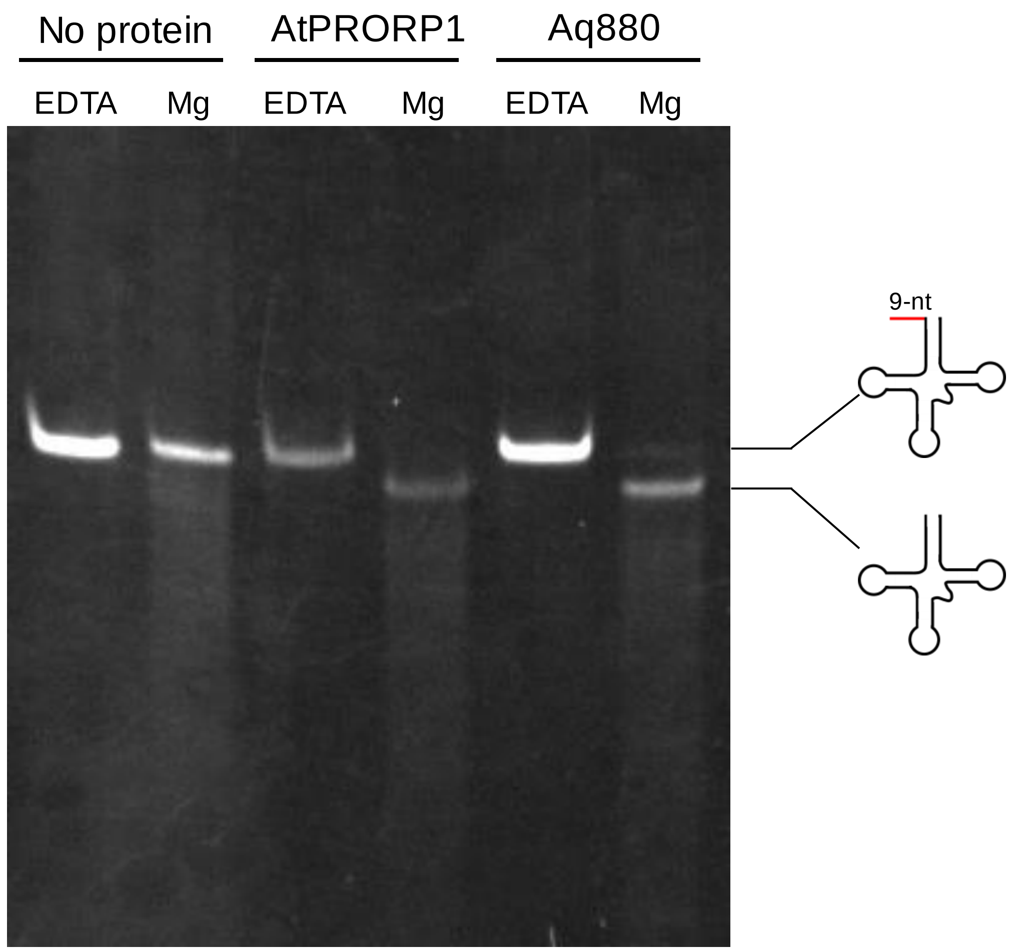

Supplementary Fig. 1. RNase P in vitro cleavage assays of Aq880. A representative TBE-urea gel of pre-tRNA processing assays with 1 μM Aq880 enzyme and 2.5 μM pre-tRNA containing 9-nt 5'-leader sequence, using experimental conditions and methods described in the Materials and Methods.

Supplementary Fig. 2.

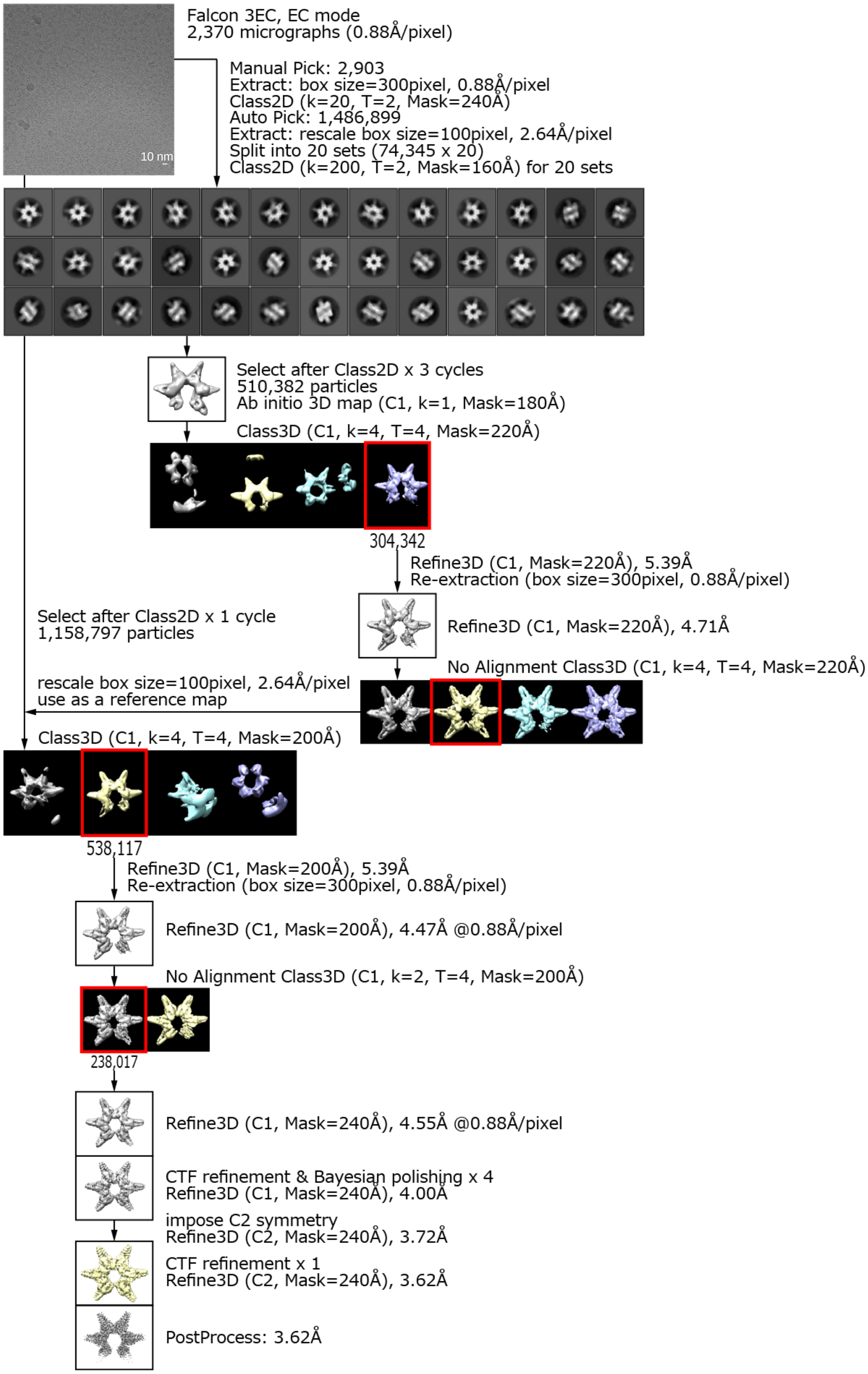

Supplementary Fig. 2. Cryo-EM data processing for Aq880. Data processing workflow used to obtain the final cryo-EM map of Aq880. The particle images of Aq880 are extracted from the same micrographs, and thus, the processing step. Please see the "Details of the cryo-EM data processing in the Supporting information for details.

Supplementary Fig. 3.

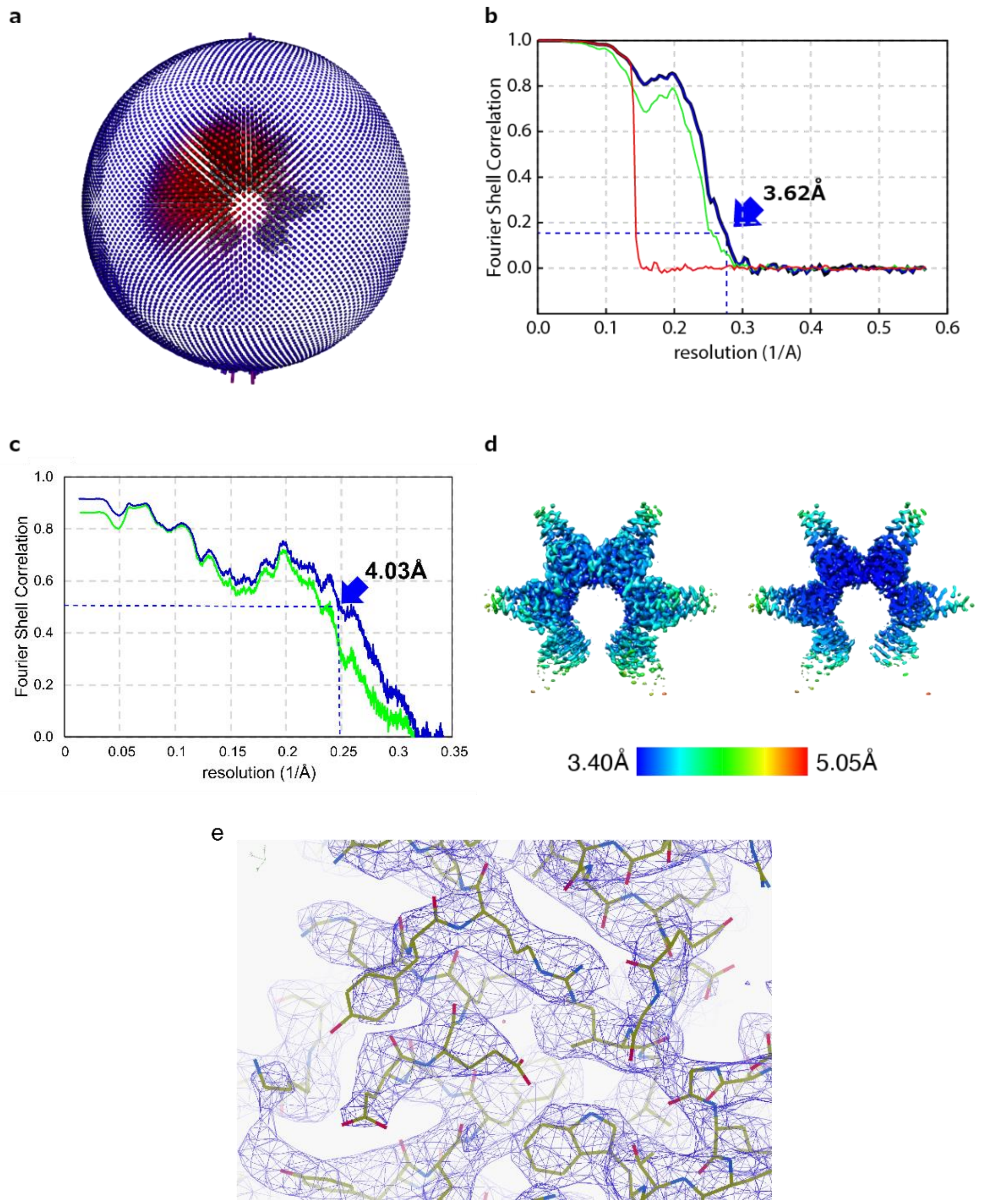

Supplementary Fig. 3. Cryo-EM data processing for Aq880. (a) Orientation distribution, (b) half-sets FSC curves, (c) map-to-model FSC curve, and (d) outer (left) and inner (right) local resolutions of the cryo-EM maps of Aq880. In the half-sets FSC curves, FSC corrected (black), FSC unmasked (green), FSC masked (blue), and corrected FSC phase randomized (red) maps are shown. (e) Representative images of the Aq880 model and surrounding electron density. Maps are displayed as mesh and contoured at 1σ.

Supplementary Fig. 4.

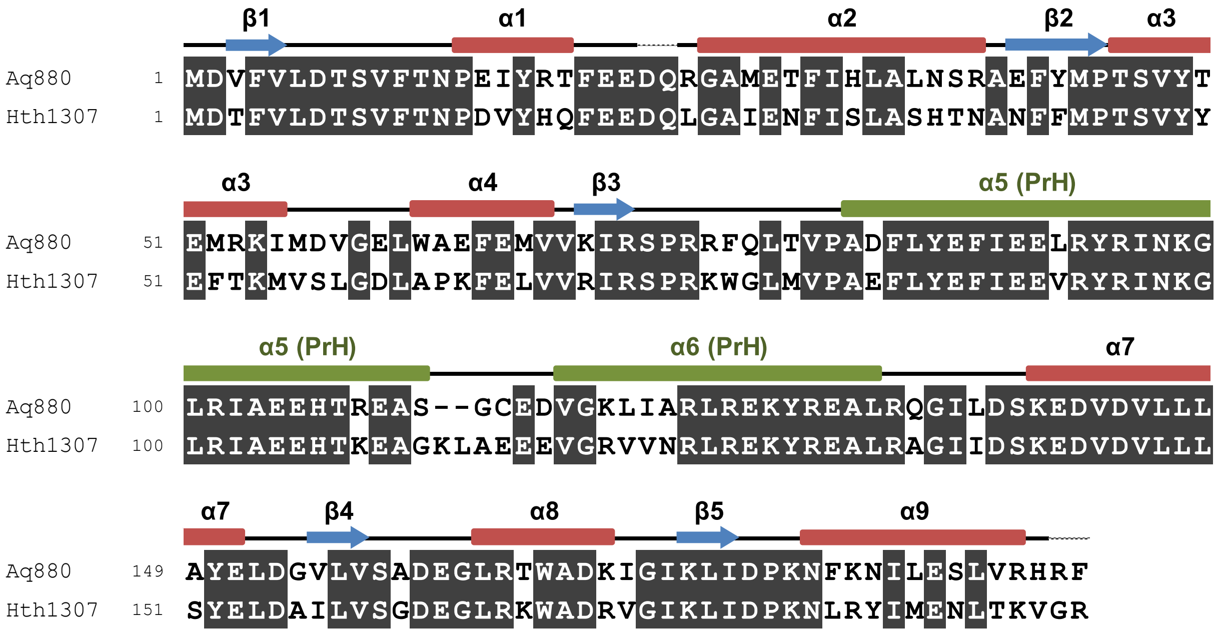

Supplementary Fig. 4. Sequence alignment of the secondary structures of Aq880 and Hth1307. The annotated secondary structure of Aq880 is indicated above the alignment (arrows: β-strands, boxes: α-helices). Identical residues are indicated by a black background. Deep green boxes indicate α-helices of PrH domain of Aq880. Dash lines indicate disorder residues.

Supplementary Fig. 5.

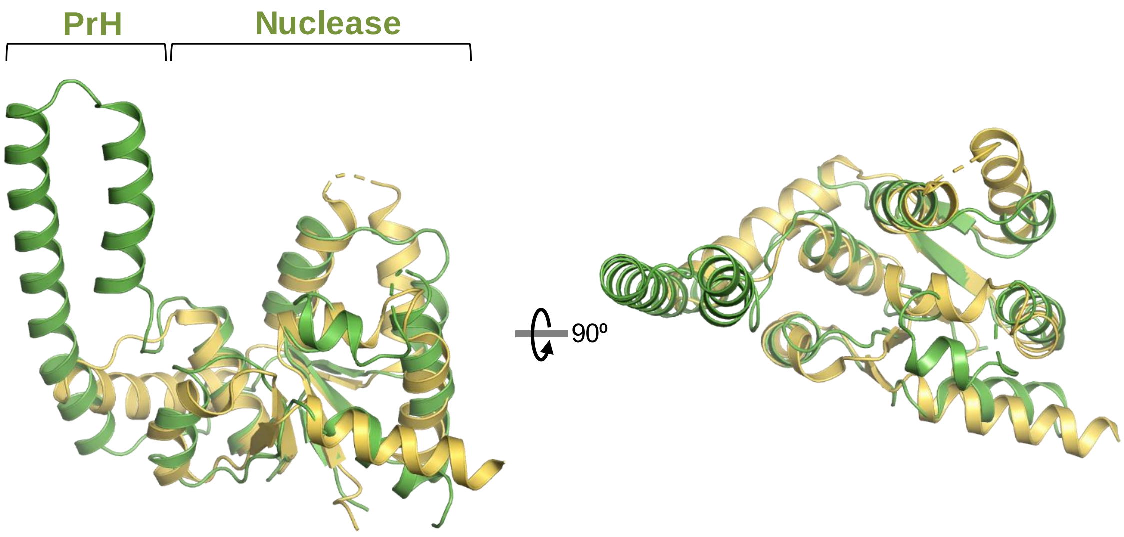

Green: Aq880
Yellow: VapC4 toxin from *Pyrococcus horikoshii*

Supplementary Fig. 5. Structural comparison between Aq880 and *Pyrococcus horikoshii* VapC4 (PhVapC4). Aq880 subunit (green) and PhVapC4 (light yellow) are shown as cartoon. The PDB ID of PhVapC4 is 5H4G. The superposition r.m.s. deviations of 3.2 Å for 139 structurally equivalent. PhVapC4 shares 11 % amino acid sequence identity with Aq880

Supplementary Fig. 6.

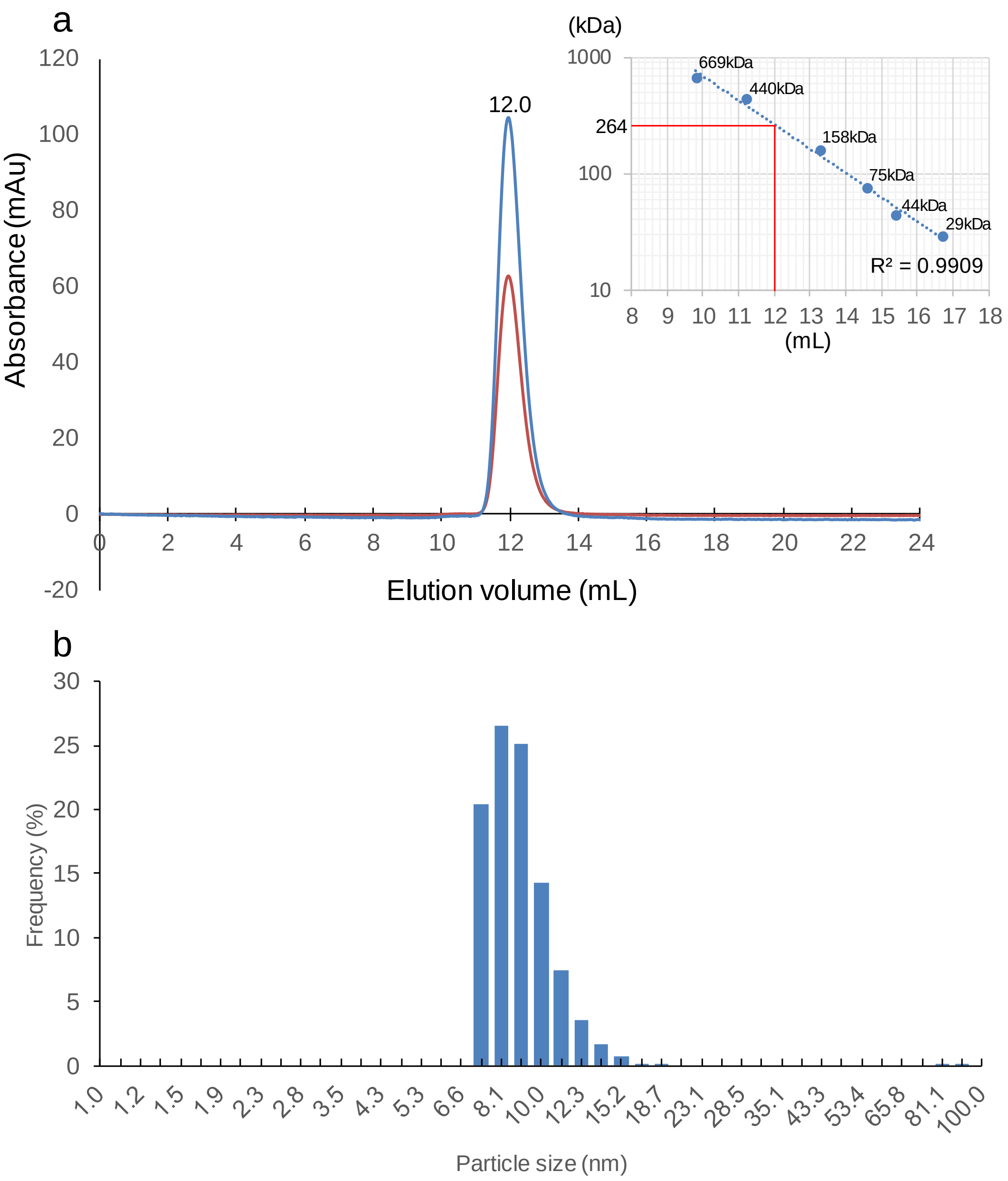

Supplementary Fig. 6. RNase P in vitro cleavage assays of Aq880. (a) Analytical gel filtration analysis of Aq880. The sample was analyzed on superdex 200 increase 10/300 GL column. The calibration curve for molecular weight determination is displayed in the upper right. Blue and red lines indicate profiles of 280 nm and 260 nm absorbance. (b) Particle size analysis of Aaq880. The sample was analyzed by using Zeta-potential & Particle size Analyzer ELSZ-2000. X and Y axis indicate particle size distribution and appearance frequency of Aq880 particles, respectively.

Supplementary Fig. 7.

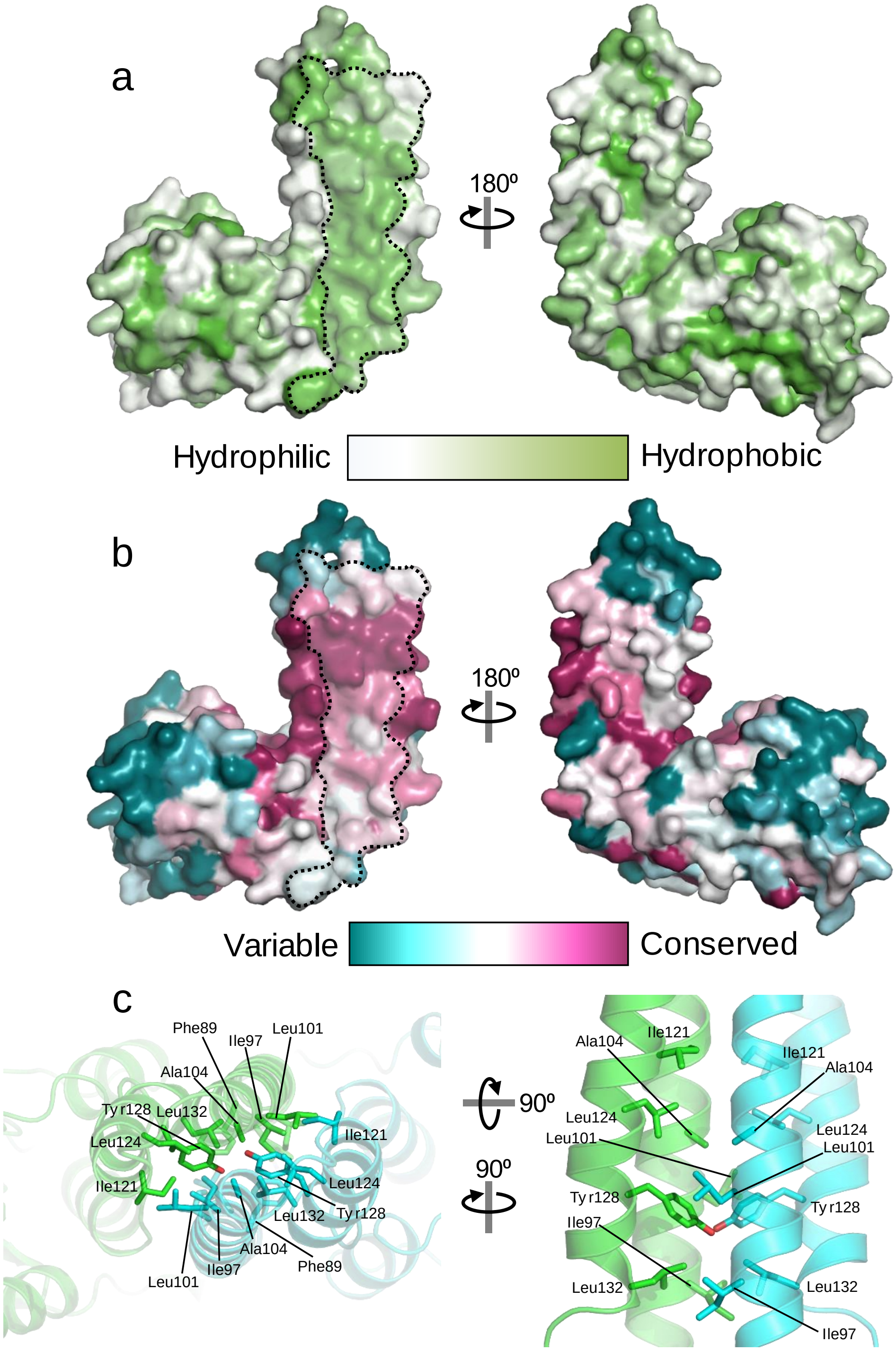

Supplementary Fig. 7. Hydrophobicity and conservation of Aq880 subunit. Dimer interfaces between two subunits indicate area surrounded by dashes (a) The Aq880 subunits are shown as surface representation with residues colored by degree of hydrophobicity, illustrating hydrophilic (white) to hydrophobic (green). (b) The Aq880 subunits are shown as surface representation with residues colored by degree of conservation, illustrating variable (deep cyan) to conserved (purple).

Supplementary Fig. 8.

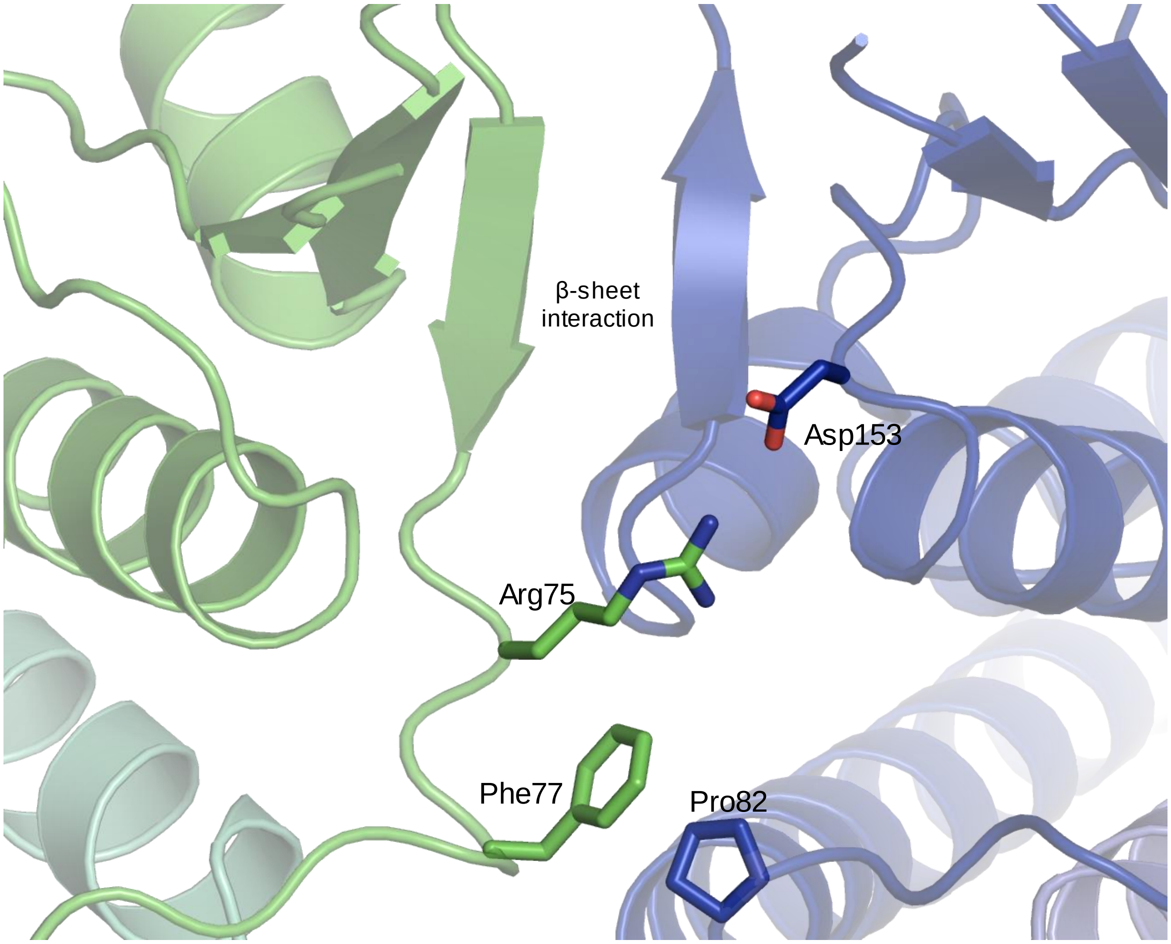

Supplementary Fig. 8. Dimer interface between two subunits. Two subunits are colored blue and green, respectively.

Supplementary Fig. 9.

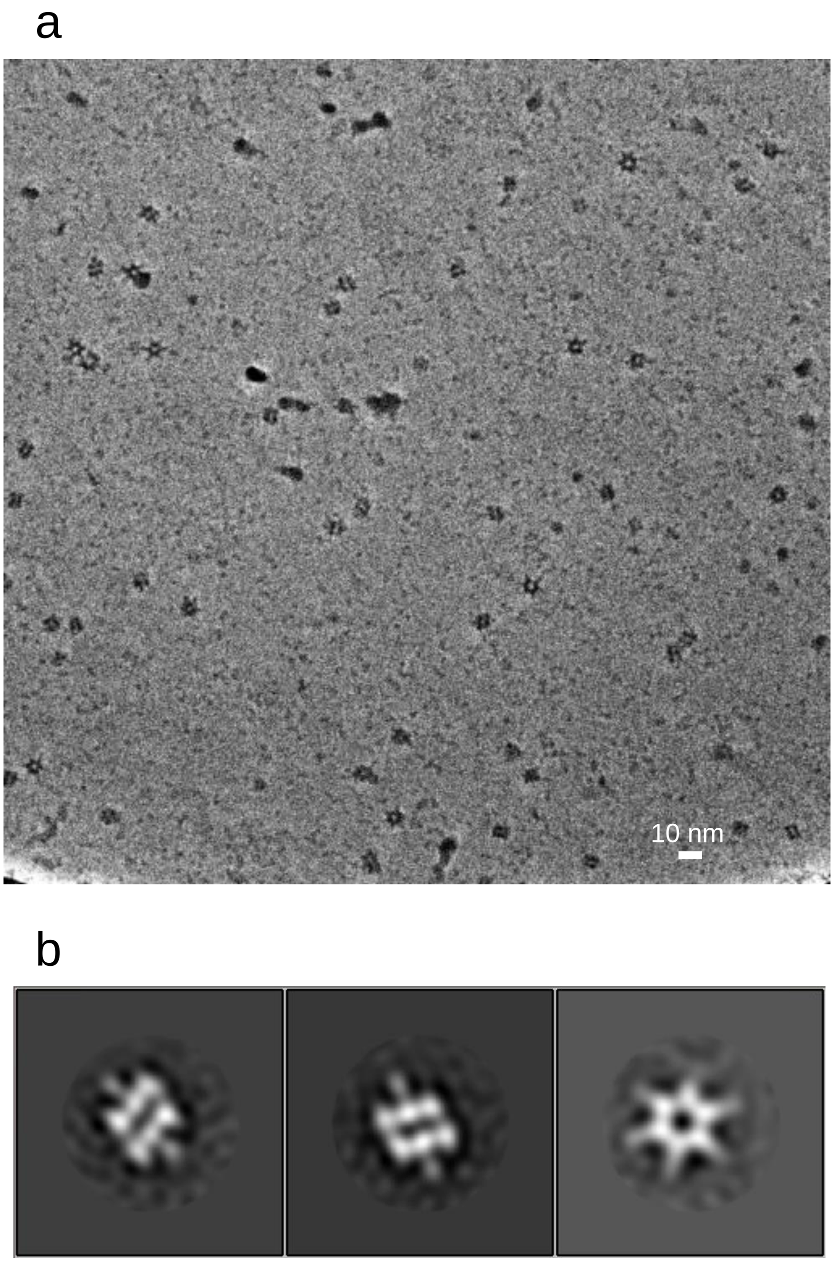

Supplementary Fig. 9. Cryo-EM analysis of Hth1307. (a) micrograph image of Hth1307. (b) 2D classification of particle images of Hth1307 (220 Å mask diameter).

Supplementary Fig. 10.

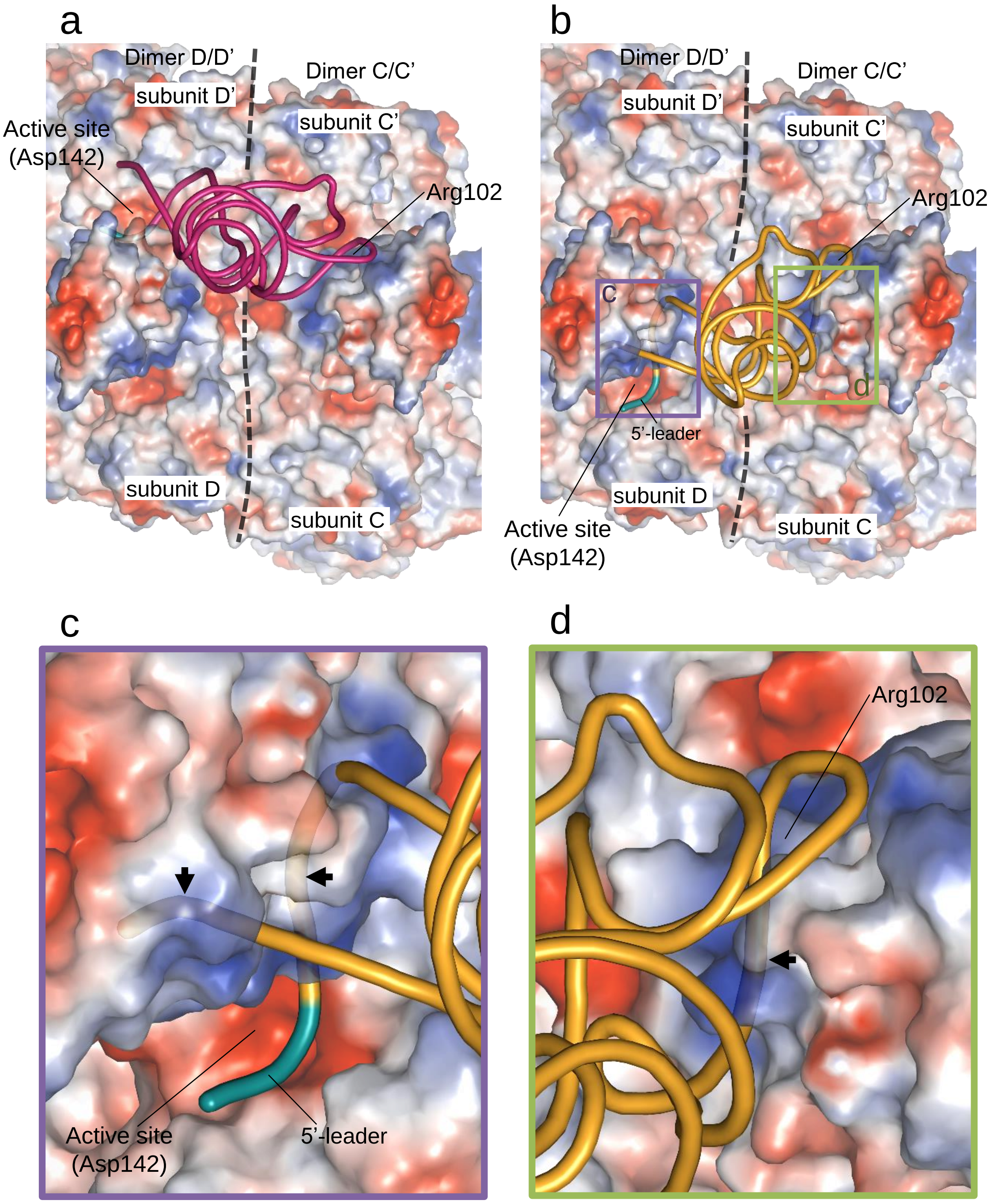

Supplementary Fig. 10 Two pre-tRNA binding candidates and one candidate causes steric hindrance. (a) The pre-tRNA molecule (red) is good binding candidates. There is no significant clash between pre-tRNA (red) and Aq880. (b) The pre-tRNA molecule (yellow) cannot fit the space due to steric hindrances. (c,d) Close-up view of steric hindrances. Black arrows indicate steric hindrances between pre-tRNA and Aq880. Aq880 is shown as surface representation with 20% transparency.

Supplementary Fig. 11.

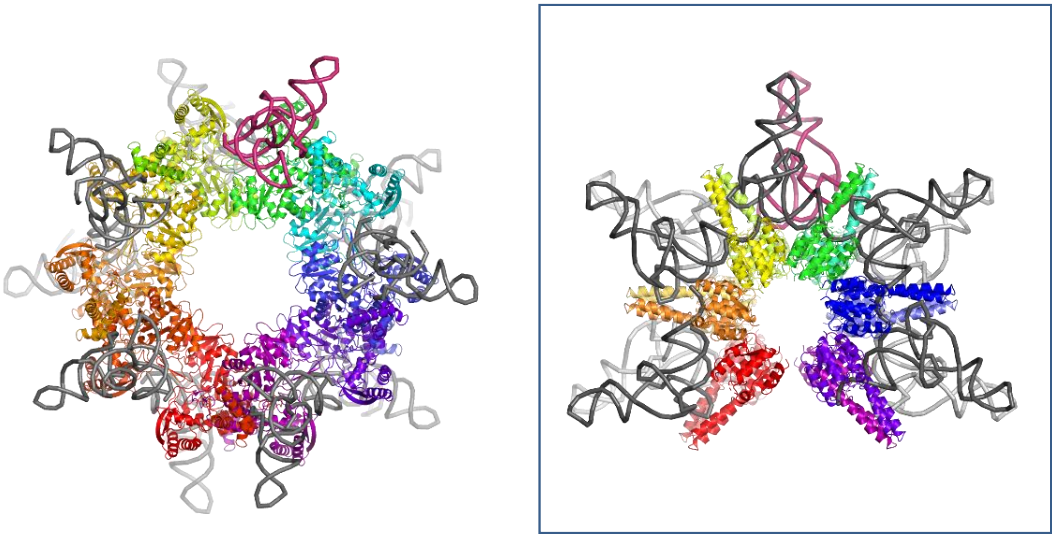

AaSelA-tRNA complex
(PDB ID: 3W1K)

Aq880-tRNA docking models
(This study)

Supplementary Fig. 11. ***A. aeolicus*** **enzymes recognize tRNA molecules by oligomerization.** AaSelA and Aq880 are shown as cartoon with rainbow colors. tRNA molecules are shown as ribbon. One representative tRNA molecule is colored warmpink.

Supplementary Fig. 12.

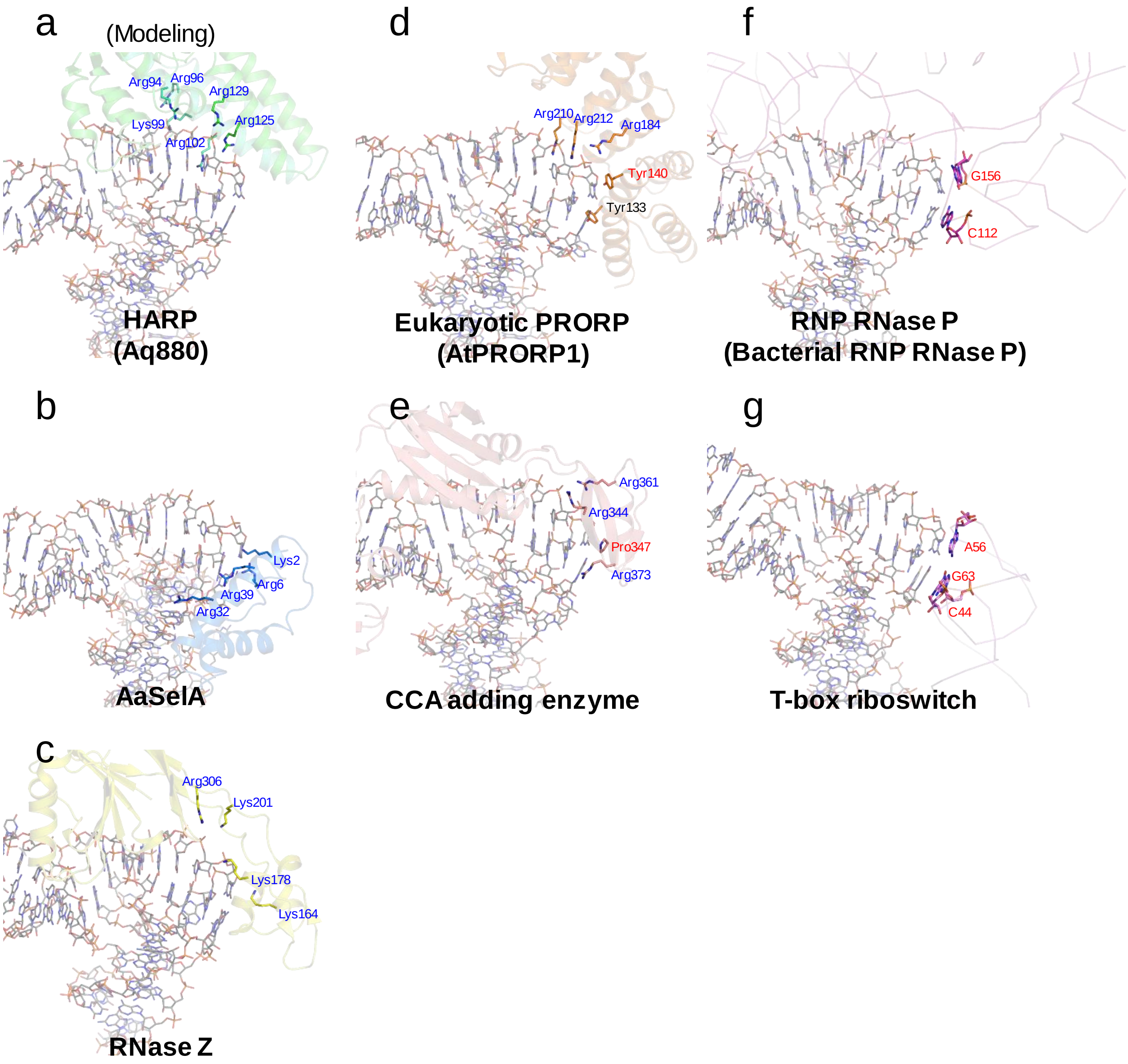

Supplementary Fig. 12 tRNA elbow recognition by proteins and RNAs. Aq880-pre-tRNA complex (a) is docking model shown in this study. PDB ID of AaSelA (b), RNase Z (c), AtPRORP1 (d), CCA adding enzyme (e), Bacterial RNP RNase P (f), and T-box riboswitch (g) are 3W1K, 4GCW, 6LVR, 1SZ1, 3Q1R, and 4LCK. Elbow binding proteins and RNAs are shown as cartoon and ribbon model, respectively, with 80% transparency. Elbow contacting residues are shown as stick model. Basic, stacking, and other interacting residues are labeled in blue, red, and black, respectively. The tRNA elbow region (gray) are shown as stick model with 40% transparency.
