# Supplementary material for: Minimal protein-only RNase P structure reveals insights into tRNA precursor recognition and catalysis
Source: J Biol Chem. 2021 Jul 31;297(3):101028. doi: 10.1016/j.jbc.2021.101028 (PMC8405995; doi:10.1016/j.jbc.2021.101028)
Supplement: Supporting data S2 [file mmc6.pdf]

Full wwPDB EM Validation Report (i)

Jun 17, 2021 – 12:50 PM JST

PDB ID	:	7F3E
EMDB ID	:	EMD-31432
Title	:	Cryo-EM structure of the minimal protein-only RNase P from Aquifex aeolicus
Deposited on	:	2021-06-16
Resolution	:	3.62 Å(reported)

This is a Full wwPDB EM Validation Report.

This report is produced by the wwPDB biocuration pipeline after annotation of the structure.

We welcome your comments at *validation@mail.wwpdb.org*
A user guide is available at
https://www.wwpdb.org/validation/2017/EMValidationReportHelp
with specific help available everywhere you see the (i) symbol.

CONFIDENTIAL VALIDATION REPORT

The following versions of software and data (see references (i)) were used in the production of this report:

MolProbity	:	4.02b-467
Percentile statistics	:	20191225.v01 (using entries in the PDB archive December 25th 2019)
Ideal geometry (proteins)	:	Engh & Huber (2001)
Ideal geometry (DNA, RNA)	:	Parkinson et al. (1996)
Validation Pipeline (wwPDB-VP)	:	2.20

1 Overall quality at a glance (i)

The following experimental techniques were used to determine the structure:
ELECTRON MICROSCOPY

The reported resolution of this entry is 3.62 Å.

Percentile scores (ranging between 0-100) for global validation metrics of the entry are shown in the following graphic. The table shows the number of entries on which the scores are based.

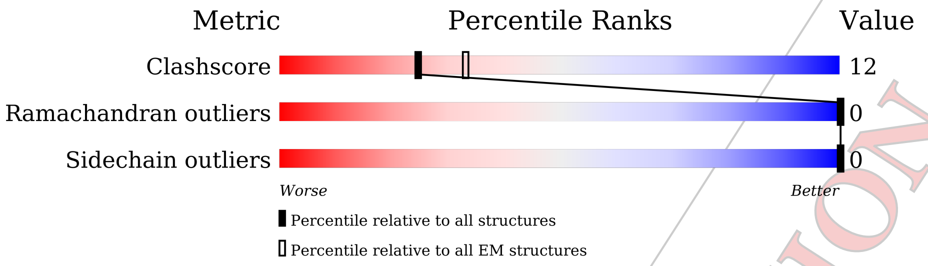

Metric	Whole archive (#Entries)	EM structures (#Entries)
Clashscore	158937	4297
Ramachandran outliers	154571	4023
Sidechain outliers	154315	3826

The table below summarises the geometric issues observed across the polymeric chains and their fit to the map. The red, orange, yellow and green segments of the bar indicate the fraction of residues that contain outliers for >=3, 2, 1 and 0 types of geometric quality criteria respectively. A grey segment represents the fraction of residues that are not modelled. The numeric value for each fraction is indicated below the corresponding segment, with a dot representing fractions <=5%

Mol	Chain	Length	Quality of chain
1	A	195	67% 29% •
1	B	195	68% 28% •
1	C	195	68% 29% •
1	D	195	68% 28% •
1	E	195	68% 29% •
1	F	195	69% 27% •
1	G	195	69% 28% •
1	H	195	67% 30% •
1	I	195	67% 29% •

Continued on next page...

CONFIDENTIAL VALIDATION REPORT

Continued from previous page...

Mol	Chain	Length	Quality of chain
1	J	195	69% 27% •
1	K	195	65% 32% •
1	L	195	66% 30% •

CONFIDENTIAL VALIDATION REPORT

2 Entry composition ⓘ

There is only 1 type of molecule in this entry. The entry contains 18564 atoms, of which 0 are hydrogens and 0 are deuteriums.

In the tables below, the AltConf column contains the number of residues with at least one atom in alternate conformation and the Trace column contains the number of residues modelled with at most 2 atoms.

- Molecule 1 is a protein called RNA-free ribonuclease P.

Mol	Chain	Residues	Atoms					AltConf	Trace
1	E	188	Total 1547	C 989	N 264	O 287	S 7	0	0
1	F	188	Total 1547	C 989	N 264	O 287	S 7	0	0
1	C	188	Total 1547	C 989	N 264	O 287	S 7	0	0
1	D	188	Total 1547	C 989	N 264	O 287	S 7	0	0
1	A	188	Total 1547	C 989	N 264	O 287	S 7	0	0
1	B	188	Total 1547	C 989	N 264	O 287	S 7	0	0
1	H	188	Total 1547	C 989	N 264	O 287	S 7	0	0
1	G	188	Total 1547	C 989	N 264	O 287	S 7	0	0
1	J	188	Total 1547	C 989	N 264	O 287	S 7	0	0
1	I	188	Total 1547	C 989	N 264	O 287	S 7	0	0
1	L	188	Total 1547	C 989	N 264	O 287	S 7	0	0
1	K	188	Total 1547	C 989	N 264	O 287	S 7	0	0

There are 36 discrepancies between the modelled and reference sequences:

Chain	Residue	Modelled	Actual	Comment	Reference
E	-2	ALA	-	expression tag	UNP O67035
E	-1	THR	-	expression tag	UNP O67035
E	0	SER	-	expression tag	UNP O67035
F	-2	ALA	-	expression tag	UNP O67035
F	-1	THR	-	expression tag	UNP O67035
F	0	SER	-	expression tag	UNP O67035

Continued on next page...

CONFIDENTIAL VALIDATION REPORT

Continued from previous page...

Chain	Residue	Modelled	Actual	Comment	Reference
C	-2	ALA	-	expression tag	UNP O67035
C	-1	THR	-	expression tag	UNP O67035
C	0	SER	-	expression tag	UNP O67035
D	-2	ALA	-	expression tag	UNP O67035
D	-1	THR	-	expression tag	UNP O67035
D	0	SER	-	expression tag	UNP O67035
A	-2	ALA	-	expression tag	UNP O67035
A	-1	THR	-	expression tag	UNP O67035
A	0	SER	-	expression tag	UNP O67035
B	-2	ALA	-	expression tag	UNP O67035
B	-1	THR	-	expression tag	UNP O67035
B	0	SER	-	expression tag	UNP O67035
H	-2	ALA	-	expression tag	UNP O67035
H	-1	THR	-	expression tag	UNP O67035
H	0	SER	-	expression tag	UNP O67035
G	-2	ALA	-	expression tag	UNP O67035
G	-1	THR	-	expression tag	UNP O67035
G	0	SER	-	expression tag	UNP O67035
J	-2	ALA	-	expression tag	UNP O67035
J	-1	THR	-	expression tag	UNP O67035
J	0	SER	-	expression tag	UNP O67035
I	-2	ALA	-	expression tag	UNP O67035
I	-1	THR	-	expression tag	UNP O67035
I	0	SER	-	expression tag	UNP O67035
L	-2	ALA	-	expression tag	UNP O67035
L	-1	THR	-	expression tag	UNP O67035
L	0	SER	-	expression tag	UNP O67035
K	-2	ALA	-	expression tag	UNP O67035
K	-1	THR	-	expression tag	UNP O67035
K	0	SER	-	expression tag	UNP O67035

CONFIDENTIAL VALIDATION REPORT

3 Residue-property plots ⓘ

These plots are drawn for all protein, RNA, DNA and oligosaccharide chains in the entry. The first graphic for a chain summarises the proportions of the various outlier classes displayed in the second graphic. The second graphic shows the sequence view annotated by issues in geometry. Residues are color-coded according to the number of geometric quality criteria for which they contain at least one outlier: green = 0, yellow = 1, orange = 2 and red = 3 or more. Stretches of 2 or more consecutive residues without any outlier are shown as a green connector. Residues present in the sample, but not in the model, are shown in grey.

- Molecule 1: RNA-free ribonuclease P

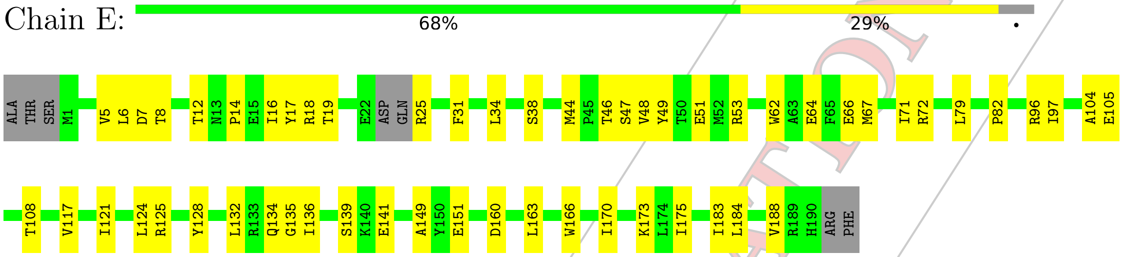

- Molecule 1: RNA-free ribonuclease P

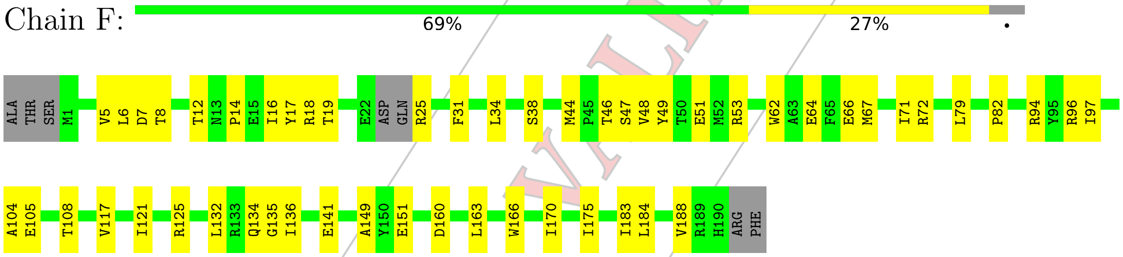

- Molecule 1: RNA-free ribonuclease P

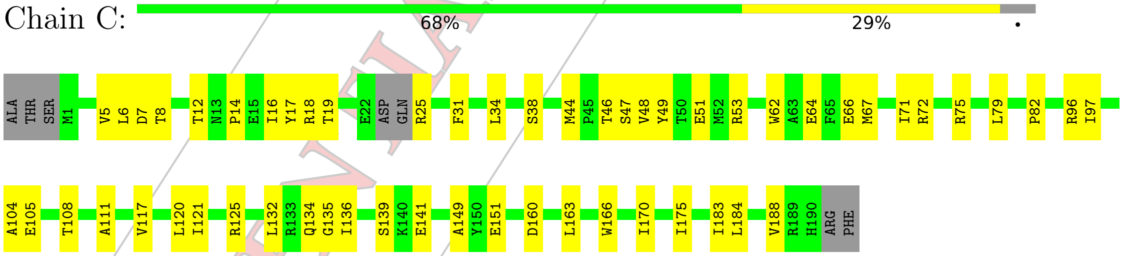

- Molecule 1: RNA-free ribonuclease P

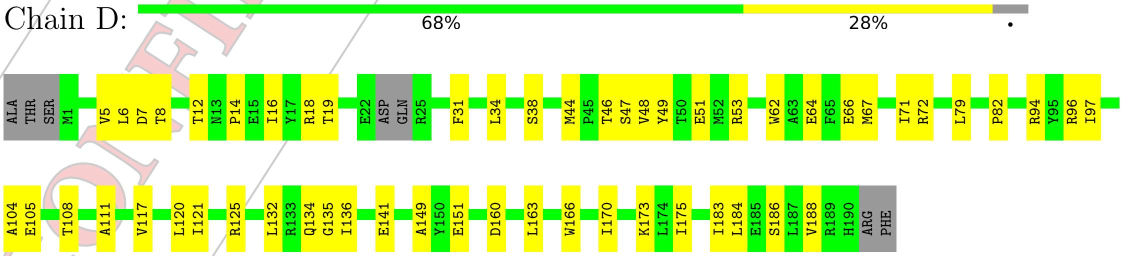

CONFIDENTIAL VALIDATION REPORT

• Molecule 1: RNA-free ribonuclease P

Chain A:

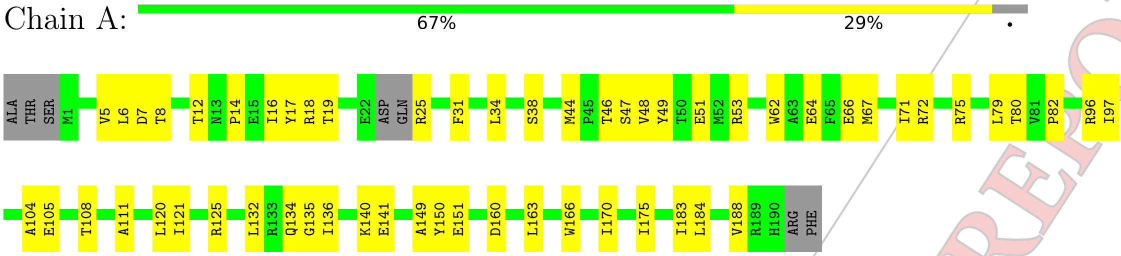

• Molecule 1: RNA-free ribonuclease P

Chain B:

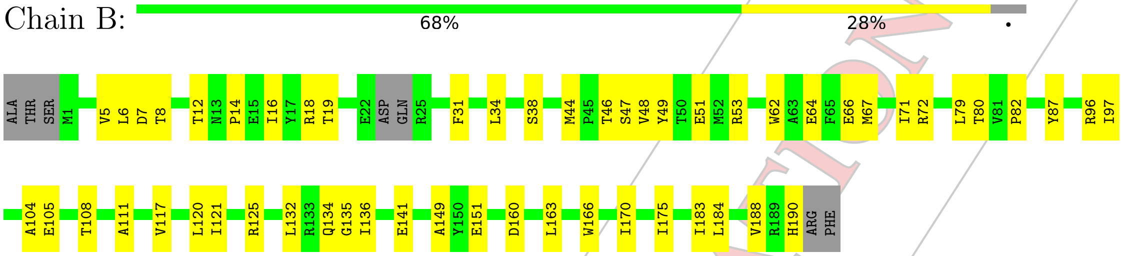

• Molecule 1: RNA-free ribonuclease P

Chain H:

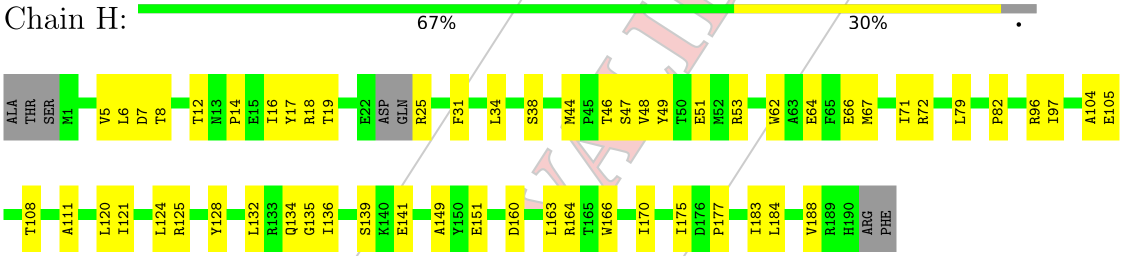

• Molecule 1: RNA-free ribonuclease P

Chain G:

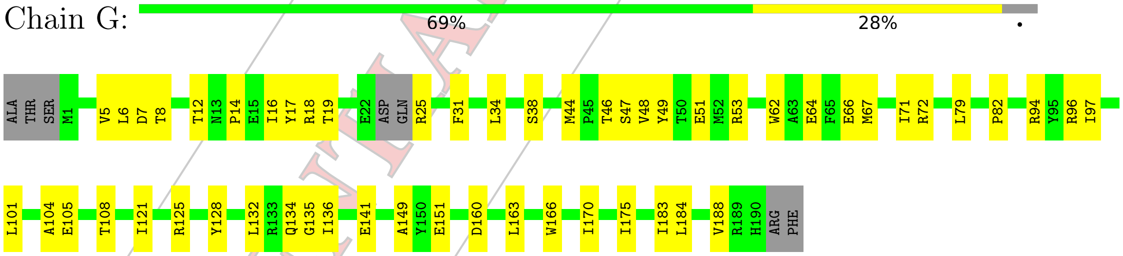

• Molecule 1: RNA-free ribonuclease P

Chain J:

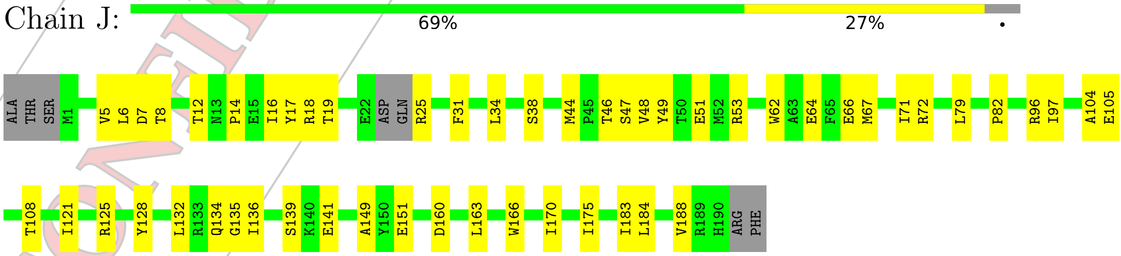

CONFIDENTIAL VALIDATION REPORT

• Molecule 1: RNA-free ribonuclease P

Chain I:

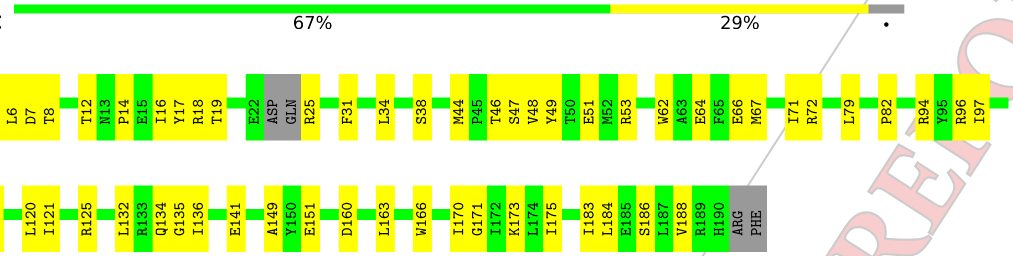

• Molecule 1: RNA-free ribonuclease P

Chain L:

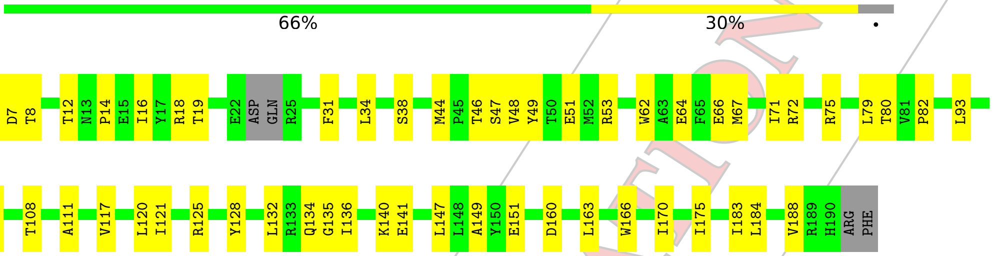

• Molecule 1: RNA-free ribonuclease P

Chain K:

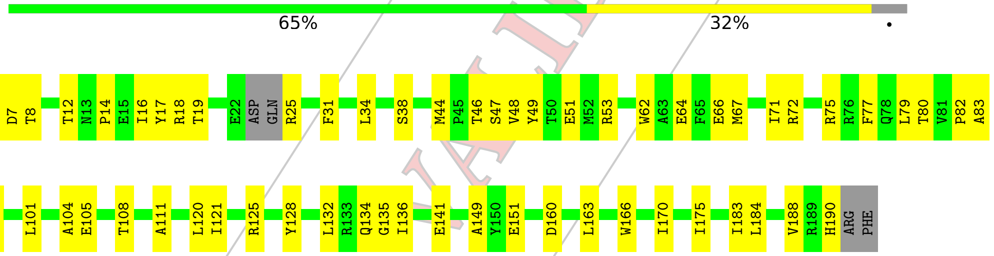

CONFIDENTIAL VALIDATION REPORT

4 Experimental information (i)

Property	Value	Source
EM reconstruction method	SINGLE PARTICLE	Depositor
Imposed symmetry	POINT, C2	Depositor
Number of particles used	238017	Depositor
Resolution determination method	FSC 0.143 CUT-OFF	Depositor
CTF correction method	PHASE FLIPPING AND AMPLITUDE CORRECTION	Depositor
Microscope	TFS TALOS	Depositor
Voltage (kV)	200	Depositor
Electron dose ($e^-/\text{Å}^2$)	50.00	Depositor
Minimum defocus (nm)	1000	Depositor
Maximum defocus (nm)	2500	Depositor
Magnification	120000	Depositor
Image detector	FEI FALCON III (4k x 4k)	Depositor

CONFIDENTIAL VALIDATION REPORT

5 Model quality (i)

5.1 Standard geometry (i)

The Z score for a bond length (or angle) is the number of standard deviations the observed value is removed from the expected value. A bond length (or angle) with $|Z| > 5$ is considered an outlier worth inspection. RMSZ is the root-mean-square of all Z scores of the bond lengths (or angles).

Mol	Chain	Bond lengths		Bond angles	
		RMSZ	#$|Z|$ >5	RMSZ	#$|Z|$ >5
1	A	0.30	0/1574	0.45	0/2119
1	B	0.30	0/1574	0.45	0/2119
1	C	0.30	0/1574	0.45	0/2119
1	D	0.30	0/1574	0.45	0/2119
1	E	0.30	0/1574	0.45	0/2119
1	F	0.30	0/1574	0.45	0/2119
1	G	0.30	0/1574	0.45	0/2119
1	H	0.30	0/1574	0.45	0/2119
1	I	0.30	0/1574	0.45	0/2119
1	J	0.30	0/1574	0.45	0/2119
1	K	0.30	0/1574	0.45	0/2119
1	L	0.30	0/1574	0.45	0/2119
All	All	0.30	0/18888	0.45	0/25428

There are no bond length outliers.

There are no bond angle outliers.

There are no chirality outliers.

There are no planarity outliers.

5.2 Too-close contacts (i)

In the following table, the Non-H and H(model) columns list the number of non-hydrogen atoms and hydrogen atoms in the chain respectively. The H(added) column lists the number of hydrogen atoms added and optimized by MolProbity. The Clashes column lists the number of clashes within the asymmetric unit, whereas Symm-Clashes lists symmetry-related clashes.

Mol	Chain	Non-H	H(model)	H(added)	Clashes	Symm-Clashes
1	A	1547	0	1556	37	0
1	B	1547	0	1556	39	0
1	C	1547	0	1556	38	0
1	D	1547	0	1556	38	0

Continued on next page...

CONFIDENTIAL VALIDATION REPORT

Continued from previous page...

Mol	Chain	Non-H	H(model)	H(added)	Clashes	Symm-Clashes
1	E	1547	0	1556	43	0
1	F	1547	0	1556	42	0
1	G	1547	0	1556	41	0
1	H	1547	0	1556	45	0
1	I	1547	0	1556	38	0
1	J	1547	0	1556	37	0
1	K	1547	0	1556	49	0
1	L	1547	0	1556	46	0
All	All	18564	0	18672	437	0

The all-atom clashscore is defined as the number of clashes found per 1000 atoms (including hydrogen atoms). The all-atom clashscore for this structure is 12.

All (437) close contacts within the same asymmetric unit are listed below, sorted by their clash magnitude.

Atom-1	Atom-2	Interatomic distance (Å)	Clash overlap (Å)
1:C:96:ARG:HH22	1:C:135:GLY:HA3	1.52	0.75
1:D:96:ARG:HH22	1:D:135:GLY:HA3	1.52	0.75
1:I:96:ARG:HH22	1:I:135:GLY:HA3	1.52	0.75
1:L:96:ARG:HH22	1:L:135:GLY:HA3	1.52	0.75
1:G:96:ARG:HH22	1:G:135:GLY:HA3	1.52	0.74
1:J:96:ARG:HH22	1:J:135:GLY:HA3	1.52	0.74
1:E:96:ARG:HH22	1:E:135:GLY:HA3	1.52	0.74
1:H:96:ARG:HH22	1:H:135:GLY:HA3	1.52	0.74
1:F:96:ARG:HH22	1:F:135:GLY:HA3	1.52	0.74
1:J:67:MET:SD	1:L:183:ILE:HG23	2.27	0.74
1:A:96:ARG:HH22	1:A:135:GLY:HA3	1.52	0.74
1:B:96:ARG:HH22	1:B:135:GLY:HA3	1.52	0.73
1:K:96:ARG:HH22	1:K:135:GLY:HA3	1.52	0.72
1:E:183:ILE:HG23	1:G:67:MET:SD	2.31	0.69
1:H:34:LEU:HB3	1:H:184:LEU:HD22	1.75	0.69
1:D:16:ILE:HA	1:D:19:THR:HG22	1.75	0.69
1:A:16:ILE:HA	1:A:19:THR:HG22	1.75	0.69
1:I:16:ILE:HA	1:I:19:THR:HG22	1.75	0.69
1:F:16:ILE:HA	1:F:19:THR:HG22	1.75	0.68
1:C:34:LEU:HB3	1:C:184:LEU:HD22	1.75	0.68
1:J:34:LEU:HB3	1:J:184:LEU:HD22	1.75	0.68
1:L:16:ILE:HA	1:L:19:THR:HG22	1.75	0.68
1:L:34:LEU:HB3	1:L:184:LEU:HD22	1.75	0.68
1:E:34:LEU:HB3	1:E:184:LEU:HD22	1.75	0.68
1:G:16:ILE:HA	1:G:19:THR:HG22	1.75	0.68

Continued on next page...

CONFIDENTIAL VALIDATION REPORT

Continued from previous page...

Atom-1	Atom-2	Interatomic distance (Å)	Clash overlap (Å)
1:K:16:ILE:HA	1:K:19:THR:HG22	1.75	0.68
1:B:16:ILE:HA	1:B:19:THR:HG22	1.75	0.68
1:J:16:ILE:HA	1:J:19:THR:HG22	1.75	0.68
1:C:16:ILE:HA	1:C:19:THR:HG22	1.75	0.68
1:G:34:LEU:HB3	1:G:184:LEU:HD22	1.75	0.68
1:B:34:LEU:HB3	1:B:184:LEU:HD22	1.75	0.68
1:E:16:ILE:HA	1:E:19:THR:HG22	1.75	0.67
1:I:34:LEU:HB3	1:I:184:LEU:HD22	1.75	0.67
1:A:34:LEU:HB3	1:A:184:LEU:HD22	1.75	0.67
1:D:34:LEU:HB3	1:D:184:LEU:HD22	1.75	0.67
1:K:34:LEU:HB3	1:K:184:LEU:HD22	1.75	0.67
1:F:46:THR:HG22	1:F:71:ILE:HG22	1.77	0.67
1:H:16:ILE:HA	1:H:19:THR:HG22	1.75	0.67
1:K:46:THR:HG22	1:K:71:ILE:HG22	1.77	0.66
1:G:46:THR:HG22	1:G:71:ILE:HG22	1.77	0.66
1:B:46:THR:HG22	1:B:71:ILE:HG22	1.77	0.66
1:F:34:LEU:HB3	1:F:184:LEU:HD22	1.75	0.66
1:A:46:THR:HG22	1:A:71:ILE:HG22	1.77	0.66
1:G:49:TYR:OH	1:G:53:ARG:NH1	2.29	0.65
1:C:49:TYR:OH	1:C:53:ARG:NH1	2.29	0.65
1:B:49:TYR:OH	1:B:53:ARG:NH1	2.29	0.65
1:H:46:THR:HG22	1:H:71:ILE:HG22	1.77	0.65
1:J:46:THR:HG22	1:J:71:ILE:HG22	1.77	0.65
1:J:49:TYR:OH	1:J:53:ARG:NH1	2.29	0.65
1:K:49:TYR:OH	1:K:53:ARG:NH1	2.29	0.65
1:D:46:THR:HG22	1:D:71:ILE:HG22	1.77	0.65
1:I:46:THR:HG22	1:I:71:ILE:HG22	1.77	0.65
1:L:49:TYR:OH	1:L:53:ARG:NH1	2.29	0.65
1:A:49:TYR:OH	1:A:53:ARG:NH1	2.30	0.65
1:H:49:TYR:OH	1:H:53:ARG:NH1	2.30	0.65
1:F:49:TYR:OH	1:F:53:ARG:NH1	2.29	0.64
1:E:46:THR:HG22	1:E:71:ILE:HG22	1.77	0.64
1:D:49:TYR:OH	1:D:53:ARG:NH1	2.29	0.64
1:F:67:MET:SD	1:H:183:ILE:HG23	2.37	0.64
1:C:46:THR:HG22	1:C:71:ILE:HG22	1.77	0.64
1:E:49:TYR:OH	1:E:53:ARG:NH1	2.29	0.64
1:L:46:THR:HG22	1:L:71:ILE:HG22	1.78	0.64
1:B:190:HIS:O	1:L:75:ARG:NH1	2.32	0.63
1:I:49:TYR:OH	1:I:53:ARG:NH1	2.29	0.63
1:K:82:PRO:HD2	1:K:170:ILE:HD11	1.81	0.63
1:L:82:PRO:HD2	1:L:170:ILE:HD11	1.81	0.62

Continued on next page...

CONFIDENTIAL VALIDATION REPORT

Continued from previous page...

Atom-1	Atom-2	Interatomic distance (Å)	Clash overlap (Å)
1:E:82:PRO:HD2	1:E:170:ILE:HD11	1.81	0.62
1:D:82:PRO:HD2	1:D:170:ILE:HD11	1.81	0.62
1:I:82:PRO:HD2	1:I:170:ILE:HD11	1.81	0.62
1:H:82:PRO:HD2	1:H:170:ILE:HD11	1.81	0.62
1:J:82:PRO:HD2	1:J:170:ILE:HD11	1.81	0.62
1:E:67:MET:SD	1:C:183:ILE:HG23	2.40	0.62
1:C:82:PRO:HD2	1:C:170:ILE:HD11	1.81	0.62
1:A:82:PRO:HD2	1:A:170:ILE:HD11	1.81	0.61
1:B:82:PRO:HD2	1:B:170:ILE:HD11	1.81	0.61
1:G:82:PRO:HD2	1:G:170:ILE:HD11	1.81	0.61
1:H:67:MET:SD	1:J:183:ILE:HG23	2.41	0.61
1:F:82:PRO:HD2	1:F:170:ILE:HD11	1.81	0.61
1:H:125:ARG:HH22	1:G:105:GLU:CD	2.05	0.60
1:L:147:LEU:CD1	1:K:83:ALA:HB1	2.33	0.59
1:A:80:THR:HB	1:B:80:THR:HB	1.84	0.59
1:I:171:GLY:HA3	1:K:75:ARG:HG2	1.84	0.58
1:E:125:ARG:HH22	1:F:105:GLU:CD	2.07	0.58
1:G:183:ILE:HG23	1:I:67:MET:SD	2.43	0.58
1:L:140:LYS:NZ	1:K:87:TYR:OH	2.31	0.57
1:L:108:THR:HG22	1:K:108:THR:HG22	1.86	0.57
1:H:5:VAL:HG21	1:H:149:ALA:HA	1.87	0.57
1:F:7:ASP:OD1	1:F:8:THR:N	2.38	0.56
1:C:67:MET:SD	1:A:183:ILE:HG23	2.46	0.56
1:D:5:VAL:HG21	1:D:149:ALA:HA	1.87	0.56
1:B:5:VAL:HG21	1:B:149:ALA:HA	1.87	0.56
1:J:5:VAL:HG21	1:J:149:ALA:HA	1.88	0.56
1:I:5:VAL:HG21	1:I:149:ALA:HA	1.87	0.56
1:K:5:VAL:HG21	1:K:149:ALA:HA	1.87	0.56
1:K:7:ASP:OD1	1:K:8:THR:N	2.38	0.56
1:B:7:ASP:OD1	1:B:8:THR:N	2.38	0.56
1:J:7:ASP:OD1	1:J:8:THR:N	2.38	0.56
1:E:5:VAL:HG21	1:E:149:ALA:HA	1.87	0.56
1:D:7:ASP:OD1	1:D:8:THR:N	2.38	0.56
1:E:183:ILE:O	1:G:67:MET:HE1	2.06	0.56
1:F:5:VAL:HG21	1:F:149:ALA:HA	1.88	0.55
1:A:5:VAL:HG21	1:A:149:ALA:HA	1.87	0.55
1:A:47:SER:O	1:A:51:GLU:HG2	2.07	0.55
1:E:47:SER:O	1:E:51:GLU:HG2	2.07	0.55
1:F:47:SER:O	1:F:51:GLU:HG2	2.07	0.55
1:C:5:VAL:HG21	1:C:149:ALA:HA	1.87	0.55
1:J:139:SER:HB3	1:I:94:ARG:NH1	2.22	0.55

Continued on next page...

CONFIDENTIAL VALIDATION REPORT

Continued from previous page...

Atom-1	Atom-2	Interatomic distance (Å)	Clash overlap (Å)
1:I:7:ASP:OD1	1:I:8:THR:N	2.38	0.55
1:I:47:SER:O	1:I:51:GLU:HG2	2.07	0.55
1:L:5:VAL:HG21	1:L:149:ALA:HA	1.87	0.55
1:L:47:SER:O	1:L:51:GLU:HG2	2.07	0.55
1:K:47:SER:O	1:K:51:GLU:HG2	2.07	0.55
1:G:5:VAL:HG21	1:G:149:ALA:HA	1.87	0.55
1:L:7:ASP:OD1	1:L:8:THR:N	2.38	0.55
1:C:47:SER:O	1:C:51:GLU:HG2	2.07	0.55
1:B:47:SER:O	1:B:51:GLU:HG2	2.07	0.55
1:G:7:ASP:OD1	1:G:8:THR:N	2.38	0.55
1:C:7:ASP:OD1	1:C:8:THR:N	2.38	0.54
1:G:47:SER:O	1:G:51:GLU:HG2	2.07	0.54
1:J:47:SER:O	1:J:51:GLU:HG2	2.07	0.54
1:D:47:SER:O	1:D:51:GLU:HG2	2.07	0.54
1:H:47:SER:O	1:H:51:GLU:HG2	2.07	0.54
1:D:121:ILE:O	1:D:125:ARG:HG2	2.07	0.54
1:A:7:ASP:OD1	1:A:8:THR:N	2.38	0.54
1:G:121:ILE:O	1:G:125:ARG:HG2	2.07	0.54
1:F:121:ILE:O	1:F:125:ARG:HG2	2.07	0.54
1:A:121:ILE:O	1:A:125:ARG:HG2	2.07	0.54
1:I:136:ILE:HG22	1:I:166:TRP:HE1	1.73	0.54
1:K:121:ILE:O	1:K:125:ARG:HG2	2.07	0.54
1:C:121:ILE:O	1:C:125:ARG:HG2	2.07	0.54
1:B:121:ILE:O	1:B:125:ARG:HG2	2.07	0.54
1:E:121:ILE:O	1:E:125:ARG:HG2	2.07	0.54
1:E:136:ILE:HG22	1:E:166:TRP:HE1	1.73	0.54
1:J:67:MET:HE3	1:L:183:ILE:O	2.08	0.54
1:E:125:ARG:HH12	1:F:105:GLU:CD	2.10	0.54
1:J:121:ILE:O	1:J:125:ARG:HG2	2.07	0.54
1:I:121:ILE:O	1:I:125:ARG:HG2	2.07	0.54
1:H:121:ILE:O	1:H:125:ARG:HG2	2.07	0.54
1:H:136:ILE:HG22	1:H:166:TRP:HE1	1.73	0.53
1:E:7:ASP:OD1	1:E:8:THR:N	2.38	0.53
1:A:136:ILE:HG22	1:A:166:TRP:HE1	1.73	0.53
1:D:136:ILE:HG22	1:D:166:TRP:HE1	1.73	0.53
1:B:136:ILE:HG22	1:B:166:TRP:HE1	1.73	0.53
1:L:121:ILE:O	1:L:125:ARG:HG2	2.07	0.53
1:L:136:ILE:HG22	1:L:166:TRP:HE1	1.73	0.53
1:G:136:ILE:HG22	1:G:166:TRP:HE1	1.73	0.53
1:C:136:ILE:HG22	1:C:166:TRP:HE1	1.73	0.53
1:H:14:PRO:O	1:H:18:ARG:HG3	2.09	0.53

Continued on next page...

CONFIDENTIAL VALIDATION REPORT

Continued from previous page...

Atom-1	Atom-2	Interatomic distance (Å)	Clash overlap (Å)
1:B:14:PRO:O	1:B:18:ARG:HG3	2.09	0.53
1:K:136:ILE:HG22	1:K:166:TRP:HE1	1.73	0.53
1:I:14:PRO:O	1:I:18:ARG:HG3	2.09	0.53
1:E:14:PRO:O	1:E:18:ARG:HG3	2.09	0.53
1:G:14:PRO:O	1:G:18:ARG:HG3	2.09	0.53
1:J:14:PRO:O	1:J:18:ARG:HG3	2.09	0.53
1:K:14:PRO:O	1:K:18:ARG:HG3	2.09	0.53
1:F:14:PRO:O	1:F:18:ARG:HG3	2.09	0.52
1:D:14:PRO:O	1:D:18:ARG:HG3	2.09	0.52
1:L:93:LEU:HD11	1:K:93:LEU:HD21	1.91	0.52
1:E:105:GLU:CD	1:F:125:ARG:HH22	2.12	0.52
1:F:136:ILE:HG22	1:F:166:TRP:HE1	1.73	0.52
1:H:105:GLU:CD	1:G:125:ARG:HH22	2.12	0.52
1:E:105:GLU:O	1:E:108:THR:OG1	2.24	0.52
1:L:14:PRO:O	1:L:18:ARG:HG3	2.09	0.52
1:C:14:PRO:O	1:C:18:ARG:HG3	2.09	0.52
1:J:136:ILE:HG22	1:J:166:TRP:HE1	1.73	0.52
1:A:38:SER:OG	1:A:188:VAL:HA	2.11	0.51
1:I:38:SER:OG	1:I:188:VAL:HA	2.11	0.51
1:A:14:PRO:O	1:A:18:ARG:HG3	2.09	0.51
1:A:140:LYS:NZ	1:B:87:TYR:OH	2.40	0.51
1:C:38:SER:OG	1:C:188:VAL:HA	2.11	0.51
1:C:79:LEU:HD21	1:C:151:GLU:HB3	1.93	0.51
1:B:38:SER:OG	1:B:188:VAL:HA	2.11	0.51
1:G:38:SER:OG	1:G:188:VAL:HA	2.11	0.51
1:F:38:SER:OG	1:F:188:VAL:HA	2.11	0.51
1:C:139:SER:HB3	1:D:94:ARG:NH1	2.25	0.51
1:A:79:LEU:HD21	1:A:151:GLU:HB3	1.93	0.51
1:L:147:LEU:HD11	1:K:83:ALA:HB1	1.93	0.51
1:F:79:LEU:HD21	1:F:151:GLU:HB3	1.93	0.50
1:D:38:SER:OG	1:D:188:VAL:HA	2.11	0.50
1:E:67:MET:HE1	1:C:183:ILE:O	2.11	0.50
1:L:38:SER:OG	1:L:188:VAL:HA	2.11	0.50
1:L:79:LEU:HD21	1:L:151:GLU:HB3	1.93	0.50
1:E:105:GLU:CD	1:F:125:ARG:HH12	2.14	0.50
1:D:175:ILE:HD11	1:D:183:ILE:HD13	1.94	0.50
1:H:79:LEU:HD21	1:H:151:GLU:HB3	1.93	0.50
1:K:38:SER:OG	1:K:188:VAL:HA	2.11	0.50
1:H:175:ILE:HD11	1:H:183:ILE:HD13	1.94	0.50
1:G:79:LEU:HD21	1:G:151:GLU:HB3	1.93	0.50
1:J:79:LEU:HD21	1:J:151:GLU:HB3	1.93	0.50

Continued on next page...

CONFIDENTIAL VALIDATION REPORT

Continued from previous page...

Atom-1	Atom-2	Interatomic distance (Å)	Clash overlap (Å)
1:K:79:LEU:HD21	1:K:151:GLU:HB3	1.93	0.50
1:E:38:SER:OG	1:E:188:VAL:HA	2.11	0.50
1:B:79:LEU:HD21	1:B:151:GLU:HB3	1.93	0.50
1:H:38:SER:OG	1:H:188:VAL:HA	2.11	0.50
1:J:38:SER:OG	1:J:188:VAL:HA	2.11	0.50
1:D:79:LEU:HD21	1:D:151:GLU:HB3	1.93	0.50
1:L:175:ILE:HD11	1:L:183:ILE:HD13	1.94	0.50
1:K:105:GLU:O	1:K:108:THR:OG1	2.24	0.50
1:F:105:GLU:O	1:F:108:THR:OG1	2.24	0.50
1:K:175:ILE:HD11	1:K:183:ILE:HD13	1.94	0.50
1:E:79:LEU:HD21	1:E:151:GLU:HB3	1.93	0.49
1:E:139:SER:HB3	1:F:94:ARG:NH1	2.27	0.49
1:G:175:ILE:HD11	1:G:183:ILE:HD13	1.94	0.49
1:I:79:LEU:HD21	1:I:151:GLU:HB3	1.93	0.49
1:I:175:ILE:HD11	1:I:183:ILE:HD13	1.94	0.49
1:E:175:ILE:HD11	1:E:183:ILE:HD13	1.94	0.49
1:B:105:GLU:O	1:B:108:THR:OG1	2.24	0.49
1:C:175:ILE:HD11	1:C:183:ILE:HD13	1.94	0.49
1:H:7:ASP:OD1	1:H:8:THR:N	2.38	0.49
1:J:175:ILE:HD11	1:J:183:ILE:HD13	1.94	0.49
1:H:125:ARG:HH12	1:G:105:GLU:CD	2.15	0.49
1:E:44:MET:O	1:E:71:ILE:HA	2.13	0.49
1:K:44:MET:O	1:K:71:ILE:HA	2.13	0.49
1:G:44:MET:O	1:G:71:ILE:HA	2.13	0.48
1:F:175:ILE:HD11	1:F:183:ILE:HD13	1.94	0.48
1:D:44:MET:O	1:D:71:ILE:HA	2.13	0.48
1:I:44:MET:O	1:I:71:ILE:HA	2.13	0.48
1:F:183:ILE:HG23	1:D:67:MET:SD	2.53	0.48
1:B:175:ILE:HD11	1:B:183:ILE:HD13	1.94	0.48
1:B:160:ASP:OD1	1:B:163:LEU:N	2.47	0.48
1:H:44:MET:O	1:H:71:ILE:HA	2.13	0.48
1:H:105:GLU:O	1:H:108:THR:OG1	2.24	0.48
1:C:44:MET:O	1:C:71:ILE:HA	2.13	0.48
1:A:44:MET:O	1:A:71:ILE:HA	2.13	0.48
1:A:75:ARG:NH1	1:K:190:HIS:O	2.45	0.48
1:B:44:MET:O	1:B:71:ILE:HA	2.13	0.48
1:A:160:ASP:OD1	1:A:163:LEU:N	2.47	0.48
1:J:160:ASP:OD1	1:J:163:LEU:N	2.47	0.48
1:E:160:ASP:OD1	1:E:163:LEU:N	2.47	0.48
1:A:175:ILE:HD11	1:A:183:ILE:HD13	1.94	0.48
1:H:64:GLU:HA	1:H:67:MET:HB2	1.96	0.48

Continued on next page...

CONFIDENTIAL VALIDATION REPORT

Continued from previous page...

Atom-1	Atom-2	Interatomic distance (Å)	Clash overlap (Å)
1:I:160:ASP:OD1	1:I:163:LEU:N	2.47	0.48
1:J:44:MET:O	1:J:71:ILE:HA	2.13	0.48
1:J:64:GLU:HA	1:J:67:MET:HB2	1.96	0.48
1:D:160:ASP:OD1	1:D:163:LEU:N	2.47	0.47
1:H:160:ASP:OD1	1:H:163:LEU:N	2.47	0.47
1:E:64:GLU:HA	1:E:67:MET:HB2	1.96	0.47
1:J:125:ARG:HH12	1:I:105:GLU:CD	2.17	0.47
1:L:160:ASP:OD1	1:L:163:LEU:N	2.47	0.47
1:G:64:GLU:HA	1:G:67:MET:HB2	1.96	0.47
1:L:64:GLU:HA	1:L:67:MET:HB2	1.96	0.47
1:H:139:SER:HB3	1:G:94:ARG:NH1	2.29	0.47
1:G:160:ASP:OD1	1:G:163:LEU:N	2.47	0.47
1:I:64:GLU:HA	1:I:67:MET:HB2	1.96	0.47
1:K:160:ASP:OD1	1:K:163:LEU:N	2.47	0.47
1:F:48:VAL:HG22	1:F:141:GLU:HB2	1.97	0.47
1:A:48:VAL:HG22	1:A:141:GLU:HB2	1.97	0.47
1:F:44:MET:O	1:F:71:ILE:HA	2.13	0.47
1:F:160:ASP:OD1	1:F:163:LEU:N	2.47	0.47
1:C:48:VAL:HG22	1:C:141:GLU:HB2	1.97	0.47
1:C:64:GLU:HA	1:C:67:MET:HB2	1.96	0.47
1:C:160:ASP:OD1	1:C:163:LEU:N	2.47	0.47
1:A:64:GLU:HA	1:A:67:MET:HB2	1.96	0.47
1:G:48:VAL:HG22	1:G:141:GLU:HB2	1.97	0.47
1:I:186:SER:HB3	1:K:67:MET:HE1	1.97	0.47
1:L:48:VAL:HG22	1:L:141:GLU:HB2	1.97	0.47
1:L:80:THR:HB	1:K:80:THR:HB	1.96	0.47
1:E:48:VAL:HG22	1:E:141:GLU:HB2	1.97	0.46
1:A:105:GLU:O	1:A:108:THR:OG1	2.24	0.46
1:K:48:VAL:HG22	1:K:141:GLU:HB2	1.97	0.46
1:D:105:GLU:O	1:D:108:THR:OG1	2.24	0.46
1:A:46:THR:HG23	1:A:72:ARG:O	2.16	0.46
1:K:64:GLU:HA	1:K:67:MET:HB2	1.96	0.46
1:E:46:THR:HG23	1:E:72:ARG:O	2.16	0.46
1:J:48:VAL:HG22	1:J:141:GLU:HB2	1.97	0.46
1:L:44:MET:O	1:L:71:ILE:HA	2.13	0.46
1:L:108:THR:HG22	1:K:108:THR:CG2	2.46	0.46
1:E:124:LEU:HD22	1:F:108:THR:HG21	1.97	0.46
1:C:46:THR:HG23	1:C:72:ARG:O	2.16	0.46
1:B:46:THR:HG23	1:B:72:ARG:O	2.15	0.46
1:J:46:THR:HG23	1:J:72:ARG:O	2.15	0.46
1:F:64:GLU:HA	1:F:67:MET:HB2	1.96	0.46

Continued on next page...

CONFIDENTIAL VALIDATION REPORT

Continued from previous page...

Atom-1	Atom-2	Interatomic distance (Å)	Clash overlap (Å)
1:L:101:LEU:HB2	1:K:128:TYR:CE2	2.50	0.46
1:D:48:VAL:HG22	1:D:141:GLU:HB2	1.97	0.46
1:D:64:GLU:HA	1:D:67:MET:HB2	1.96	0.46
1:B:64:GLU:HA	1:B:67:MET:HB2	1.96	0.46
1:H:48:VAL:HG22	1:H:141:GLU:HB2	1.97	0.46
1:J:105:GLU:O	1:J:108:THR:OG1	2.24	0.46
1:I:48:VAL:HG22	1:I:141:GLU:HB2	1.97	0.46
1:G:46:THR:HG23	1:G:72:ARG:O	2.16	0.46
1:K:46:THR:HG23	1:K:72:ARG:O	2.16	0.46
1:D:46:THR:HG23	1:D:72:ARG:O	2.16	0.46
1:H:46:THR:HG23	1:H:72:ARG:O	2.15	0.46
1:I:46:THR:HG23	1:I:72:ARG:O	2.16	0.46
1:F:46:THR:HG23	1:F:72:ARG:O	2.16	0.45
1:D:186:SER:HB3	1:B:67:MET:CE	2.46	0.45
1:B:48:VAL:HG22	1:B:141:GLU:HB2	1.97	0.45
1:L:46:THR:HG23	1:L:72:ARG:O	2.16	0.45
1:A:96:ARG:NH1	1:A:134:GLN:O	2.50	0.45
1:L:93:LEU:CD1	1:K:93:LEU:HD21	2.46	0.45
1:B:96:ARG:NH1	1:B:134:GLN:O	2.50	0.45
1:B:62:TRP:O	1:B:66:GLU:HG2	2.17	0.45
1:C:62:TRP:O	1:C:66:GLU:HG2	2.17	0.45
1:C:96:ARG:NH1	1:C:134:GLN:O	2.50	0.45
1:J:8:THR:O	1:J:12:THR:HG22	2.17	0.45
1:L:62:TRP:O	1:L:66:GLU:HG2	2.17	0.45
1:A:8:THR:O	1:A:12:THR:HG22	2.17	0.45
1:A:62:TRP:O	1:A:66:GLU:HG2	2.17	0.45
1:K:8:THR:O	1:K:12:THR:HG22	2.17	0.45
1:D:62:TRP:O	1:D:66:GLU:HG2	2.17	0.45
1:D:6:LEU:HD23	1:D:6:LEU:HA	1.86	0.44
1:H:62:TRP:O	1:H:66:GLU:HG2	2.17	0.44
1:G:8:THR:O	1:G:12:THR:HG22	2.17	0.44
1:K:62:TRP:O	1:K:66:GLU:HG2	2.17	0.44
1:H:124:LEU:HD22	1:G:108:THR:HG21	1.99	0.44
1:G:6:LEU:HD11	1:G:31:PHE:HZ	1.83	0.44
1:J:104:ALA:O	1:J:108:THR:HG23	2.18	0.44
1:I:6:LEU:HD11	1:I:31:PHE:HZ	1.83	0.44
1:I:105:GLU:O	1:I:108:THR:OG1	2.24	0.44
1:L:96:ARG:NH1	1:L:134:GLN:O	2.50	0.44
1:E:62:TRP:O	1:E:66:GLU:HG2	2.17	0.44
1:D:8:THR:O	1:D:12:THR:HG22	2.17	0.44
1:H:105:GLU:CD	1:G:125:ARG:HH12	2.21	0.44

Continued on next page...

CONFIDENTIAL VALIDATION REPORT

Continued from previous page...

Atom-1	Atom-2	Interatomic distance (Å)	Clash overlap (Å)
1:L:105:GLU:O	1:L:108:THR:OG1	2.24	0.44
1:E:173:LYS:HB3	1:G:72:ARG:HG2	2.00	0.44
1:F:8:THR:O	1:F:12:THR:HG22	2.17	0.44
1:C:8:THR:O	1:C:12:THR:HG22	2.17	0.44
1:D:6:LEU:HD11	1:D:31:PHE:HZ	1.83	0.44
1:B:8:THR:O	1:B:12:THR:HG22	2.17	0.44
1:K:6:LEU:HD11	1:K:31:PHE:HZ	1.82	0.44
1:F:6:LEU:HD11	1:F:31:PHE:HZ	1.83	0.44
1:A:104:ALA:O	1:A:108:THR:HG23	2.18	0.44
1:B:6:LEU:HD11	1:B:31:PHE:HZ	1.82	0.44
1:B:104:ALA:O	1:B:108:THR:HG23	2.18	0.44
1:H:8:THR:O	1:H:12:THR:HG22	2.17	0.44
1:H:104:ALA:O	1:H:108:THR:HG23	2.18	0.44
1:I:8:THR:O	1:I:12:THR:HG22	2.17	0.44
1:I:104:ALA:O	1:I:108:THR:HG23	2.18	0.44
1:L:6:LEU:HD11	1:L:31:PHE:HZ	1.82	0.44
1:I:96:ARG:NH1	1:I:134:GLN:O	2.50	0.44
1:L:8:THR:O	1:L:12:THR:HG22	2.17	0.44
1:I:62:TRP:O	1:I:66:GLU:HG2	2.17	0.44
1:K:96:ARG:NH1	1:K:134:GLN:O	2.50	0.44
1:E:8:THR:O	1:E:12:THR:HG22	2.17	0.44
1:B:6:LEU:HD23	1:B:6:LEU:HA	1.86	0.44
1:H:96:ARG:NH1	1:H:134:GLN:O	2.50	0.44
1:G:105:GLU:O	1:G:108:THR:OG1	2.24	0.44
1:F:62:TRP:O	1:F:66:GLU:HG2	2.17	0.43
1:C:6:LEU:HD11	1:C:31:PHE:HZ	1.82	0.43
1:D:186:SER:HB3	1:B:67:MET:HE1	1.99	0.43
1:D:96:ARG:NH1	1:D:134:GLN:O	2.50	0.43
1:G:104:ALA:O	1:G:108:THR:HG23	2.18	0.43
1:E:6:LEU:HD11	1:E:31:PHE:HZ	1.83	0.43
1:L:128:TYR:CE2	1:K:101:LEU:HB2	2.53	0.43
1:G:62:TRP:O	1:G:66:GLU:HG2	2.17	0.43
1:G:96:ARG:NH1	1:G:134:GLN:O	2.50	0.43
1:L:104:ALA:O	1:L:108:THR:HG23	2.18	0.43
1:F:96:ARG:NH1	1:F:134:GLN:O	2.50	0.43
1:D:104:ALA:O	1:D:108:THR:HG23	2.18	0.43
1:H:6:LEU:HD11	1:H:31:PHE:HZ	1.83	0.43
1:J:6:LEU:HD11	1:J:31:PHE:HZ	1.82	0.43
1:F:67:MET:HE1	1:H:183:ILE:HA	2.01	0.43
1:H:97:ILE:HA	1:H:132:LEU:HD21	2.01	0.43
1:G:8:THR:HB	1:G:48:VAL:HG13	2.01	0.43

Continued on next page...

CONFIDENTIAL VALIDATION REPORT

Continued from previous page...

Atom-1	Atom-2	Interatomic distance (Å)	Clash overlap (Å)
1:J:62:TRP:O	1:J:66:GLU:HG2	2.17	0.43
1:E:96:ARG:NH1	1:E:134:GLN:O	2.50	0.43
1:E:97:ILE:HA	1:E:132:LEU:HD21	2.01	0.43
1:F:104:ALA:O	1:F:108:THR:HG23	2.18	0.43
1:C:104:ALA:O	1:C:108:THR:HG23	2.18	0.43
1:A:6:LEU:HD11	1:A:31:PHE:HZ	1.83	0.43
1:A:97:ILE:HA	1:A:132:LEU:HD21	2.01	0.43
1:H:8:THR:HB	1:H:48:VAL:HG13	2.01	0.43
1:K:8:THR:HB	1:K:48:VAL:HG13	2.01	0.43
1:K:97:ILE:HA	1:K:132:LEU:HD21	2.01	0.43
1:C:105:GLU:O	1:C:108:THR:OG1	2.24	0.43
1:F:97:ILE:HA	1:F:132:LEU:HD21	2.01	0.42
1:C:8:THR:HB	1:C:48:VAL:HG13	2.01	0.42
1:J:96:ARG:NH1	1:J:134:GLN:O	2.50	0.42
1:E:104:ALA:O	1:E:108:THR:HG23	2.18	0.42
1:F:67:MET:HE1	1:H:183:ILE:O	2.19	0.42
1:B:97:ILE:HA	1:B:132:LEU:HD21	2.01	0.42
1:H:67:MET:HE1	1:J:183:ILE:O	2.18	0.42
1:K:104:ALA:O	1:K:108:THR:HG23	2.18	0.42
1:F:8:THR:HB	1:F:48:VAL:HG13	2.01	0.42
1:D:173:LYS:HB3	1:B:72:ARG:HG2	2.02	0.42
1:H:128:TYR:CE2	1:G:101:LEU:HB2	2.54	0.42
1:J:8:THR:HB	1:J:48:VAL:HG13	2.01	0.42
1:G:97:ILE:HA	1:G:132:LEU:HD21	2.01	0.42
1:I:97:ILE:HA	1:I:132:LEU:HD21	2.01	0.42
1:A:8:THR:HB	1:A:48:VAL:HG13	2.01	0.42
1:B:8:THR:HB	1:B:48:VAL:HG13	2.01	0.42
1:L:97:ILE:HA	1:L:132:LEU:HD21	2.01	0.42
1:D:97:ILE:HA	1:D:132:LEU:HD21	2.01	0.42
1:J:97:ILE:HA	1:J:132:LEU:HD21	2.01	0.42
1:E:8:THR:HB	1:E:48:VAL:HG13	2.01	0.41
1:F:17:TYR:HH	1:F:25:ARG:N	2.19	0.41
1:J:17:TYR:HH	1:J:25:ARG:N	2.19	0.41
1:E:17:TYR:HH	1:E:25:ARG:N	2.19	0.41
1:D:8:THR:HB	1:D:48:VAL:HG13	2.01	0.41
1:I:8:THR:HB	1:I:48:VAL:HG13	2.01	0.41
1:C:97:ILE:HA	1:C:132:LEU:HD21	2.01	0.41
1:D:111:ALA:HB2	1:D:120:LEU:HD23	2.03	0.41
1:B:5:VAL:HG11	1:B:149:ALA:HB2	2.03	0.41
1:K:111:ALA:HB2	1:K:120:LEU:HD23	2.03	0.41
1:A:111:ALA:HB2	1:A:120:LEU:HD23	2.03	0.41

Continued on next page...

CONFIDENTIAL VALIDATION REPORT

Continued from previous page...

Atom-1	Atom-2	Interatomic distance (Å)	Clash overlap (Å)
1:G:17:TYR:HH	1:G:25:ARG:N	2.19	0.41
1:I:5:VAL:HG11	1:I:149:ALA:HB2	2.03	0.41
1:I:17:TYR:HH	1:I:25:ARG:N	2.19	0.41
1:F:5:VAL:HG11	1:F:149:ALA:HB2	2.03	0.41
1:C:17:TYR:HH	1:C:25:ARG:N	2.19	0.41
1:C:75:ARG:NH1	1:A:150:TYR:HE1	2.19	0.41
1:A:17:TYR:HH	1:A:25:ARG:N	2.19	0.41
1:F:117:VAL:O	1:F:121:ILE:HG12	2.21	0.41
1:C:44:MET:HE3	1:C:49:TYR:HB2	2.03	0.41
1:B:111:ALA:HB2	1:B:120:LEU:HD23	2.03	0.41
1:H:5:VAL:HG11	1:H:149:ALA:HB2	2.03	0.41
1:H:17:TYR:HH	1:H:25:ARG:N	2.19	0.41
1:I:170:ILE:O	1:K:77:PHE:HE2	2.04	0.41
1:L:8:THR:HB	1:L:48:VAL:HG13	2.01	0.41
1:K:17:TYR:HH	1:K:25:ARG:N	2.19	0.41
1:C:111:ALA:HB2	1:C:120:LEU:HD23	2.03	0.41
1:I:173:LYS:HB3	1:K:72:ARG:HG2	2.03	0.41
1:L:111:ALA:HB2	1:L:120:LEU:HD23	2.03	0.41
1:L:117:VAL:O	1:L:121:ILE:HG12	2.21	0.41
1:L:147:LEU:HD13	1:K:83:ALA:HB1	2.03	0.41
1:E:5:VAL:HG11	1:E:149:ALA:HB2	2.03	0.40
1:E:117:VAL:O	1:E:121:ILE:HG12	2.21	0.40
1:G:128:TYR:CE2	1:G:132:LEU:HD12	2.57	0.40
1:J:128:TYR:CE2	1:J:132:LEU:HD12	2.56	0.40
1:I:111:ALA:HB2	1:I:120:LEU:HD23	2.03	0.40
1:L:108:THR:CG2	1:K:108:THR:HG22	2.50	0.40
1:E:128:TYR:CE2	1:E:132:LEU:HD12	2.57	0.40
1:D:5:VAL:HG11	1:D:149:ALA:HB2	2.03	0.40
1:A:5:VAL:HG11	1:A:149:ALA:HB2	2.03	0.40
1:B:117:VAL:O	1:B:121:ILE:HG12	2.21	0.40
1:H:111:ALA:HB2	1:H:120:LEU:HD23	2.03	0.40
1:L:128:TYR:CE2	1:L:132:LEU:HD12	2.56	0.40
1:F:44:MET:HE3	1:F:49:TYR:HB2	2.04	0.40
1:K:5:VAL:HG11	1:K:149:ALA:HB2	2.03	0.40
1:K:128:TYR:CE2	1:K:132:LEU:HD12	2.57	0.40
1:C:117:VAL:O	1:C:121:ILE:HG12	2.21	0.40
1:C:125:ARG:HH22	1:D:105:GLU:CD	2.24	0.40
1:B:44:MET:HE3	1:B:49:TYR:HB2	2.03	0.40
1:H:164:ARG:NH1	1:H:177:PRO:HD3	2.37	0.40
1:J:44:MET:HE3	1:J:49:TYR:HB2	2.04	0.40
1:D:117:VAL:O	1:D:121:ILE:HG12	2.21	0.40

Continued on next page...

CONFIDENTIAL VALIDATION REPORT

Continued from previous page...

Atom-1	Atom-2	Interatomic distance (Å)	Clash overlap (Å)
1:A:44:MET:HE3	1:A:49:TYR:HB2	2.04	0.40
1:H:44:MET:HE3	1:H:49:TYR:HB2	2.04	0.40

There are no symmetry-related clashes.

5.3 Torsion angles (i)

5.3.1 Protein backbone (i)

In the following table, the Percentiles column shows the percent Ramachandran outliers of the chain as a percentile score with respect to all PDB entries followed by that with respect to all EM entries.

The Analysed column shows the number of residues for which the backbone conformation was analysed, and the total number of residues.

Mol	Chain	Analysed	Favoured	Allowed	Outliers	Percentiles	
1	A	184/195 (94%)	177 (96%)	7 (4%)	0	100	100
1	B	184/195 (94%)	177 (96%)	7 (4%)	0	100	100
1	C	184/195 (94%)	178 (97%)	6 (3%)	0	100	100
1	D	184/195 (94%)	177 (96%)	7 (4%)	0	100	100
1	E	184/195 (94%)	177 (96%)	7 (4%)	0	100	100
1	F	184/195 (94%)	177 (96%)	7 (4%)	0	100	100
1	G	184/195 (94%)	177 (96%)	7 (4%)	0	100	100
1	H	184/195 (94%)	177 (96%)	7 (4%)	0	100	100
1	I	184/195 (94%)	177 (96%)	7 (4%)	0	100	100
1	J	184/195 (94%)	177 (96%)	7 (4%)	0	100	100
1	K	184/195 (94%)	177 (96%)	7 (4%)	0	100	100
1	L	184/195 (94%)	177 (96%)	7 (4%)	0	100	100
All	All	2208/2340 (94%)	2125 (96%)	83 (4%)	0	100	100

There are no Ramachandran outliers to report.

CONFIDENTIAL VALIDATION REPORT

5.3.2 Protein sidechains (i)

In the following table, the Percentiles column shows the percent sidechain outliers of the chain as a percentile score with respect to all PDB entries followed by that with respect to all EM entries.

The Analysed column shows the number of residues for which the sidechain conformation was analysed, and the total number of residues.

Mol	Chain	Analysed	Rotameric	Outliers	Percentiles	
1	A	167/173 (96%)	167 (100%)	0	100	100
1	B	167/173 (96%)	167 (100%)	0	100	100
1	C	167/173 (96%)	167 (100%)	0	100	100
1	D	167/173 (96%)	167 (100%)	0	100	100
1	E	167/173 (96%)	167 (100%)	0	100	100
1	F	167/173 (96%)	167 (100%)	0	100	100
1	G	167/173 (96%)	167 (100%)	0	100	100
1	H	167/173 (96%)	167 (100%)	0	100	100
1	I	167/173 (96%)	167 (100%)	0	100	100
1	J	167/173 (96%)	167 (100%)	0	100	100
1	K	167/173 (96%)	167 (100%)	0	100	100
1	L	167/173 (96%)	167 (100%)	0	100	100
All	All	2004/2076 (96%)	2004 (100%)	0	100	100

There are no protein residues with a non-rotameric sidechain to report.

Sometimes sidechains can be flipped to improve hydrogen bonding and reduce clashes. There are no such sidechains identified.

5.3.3 RNA (i)

There are no RNA molecules in this entry.

5.4 Non-standard residues in protein, DNA, RNA chains (i)

There are no non-standard protein/DNA/RNA residues in this entry.

5.5 Carbohydrates (i)

There are no monosaccharides in this entry.

CONFIDENTIAL VALIDATION REPORT

5.6 Ligand geometry (i)

There are no ligands in this entry.

5.7 Other polymers (i)

There are no such residues in this entry.

5.8 Polymer linkage issues (i)

There are no chain breaks in this entry.

CONFIDENTIAL VALIDATION REPORT
